# Changes in Gut Microbial Community of Pig Feces in Response to Different Dietary Animal Protein Media

**DOI:** 10.4014/jmb.2003.03021

**Published:** 2020-05-31

**Authors:** Yujeong Jeong, Jongbin Park, Eun Bae Kim

**Affiliations:** 1 Department of Applied Animal Science, College of Animal Life Science, Kangwon National University, Chuncheon 24341, Republic of Korea; 2 Department of Animal Life Science, College of Animal Life Science, Kangwon National University, Chuncheon 24341, Republic of Korea

**Keywords:** Protein, protein utilizing bacteria, meat, milk, fecal contamination, gut microbiome

## Abstract

Beef, pork, chicken and milk are considered representative protein sources in the human diet. Since the digestion of protein is important, the role of intestinal microflora is also important. Despite this, the pure effects of meat and milk intake on the microbiome are yet to be fully elucidated. To evaluate the effect of beef, pork, chicken and milk on intestinal microflora, we observed changes in the microbiome in response to different types of dietary animal proteins in vitro*.* Feces were collected from five 6-week-old pigs. The suspensions were pooled and inoculated into four different media containing beef, pork, chicken, or skim milk powder in distilled water. Changes in microbial communities were analyzed using 16S rRNA sequencing. The feces alone had the highest microbial alpha diversity. Among the treatment groups, beef showed the highest microbial diversity, followed by pork, chicken, and milk. The three dominant phyla were Proteobacteria, Firmicutes, and Bacteroidetes in all the groups. The most abundant genera in beef, pork, and chicken were *Rummeliibacillus*, *Clostridium*, and *Phascolarctobacterium*, whereas milk was enriched with *Streptococcus*, *Lactobacillus*, and *Enterococcus*. Aerobic bacteria decreased while anaerobic and facultative anaerobic bacteria increased in protein-rich nutrients. Functional gene groups were found to be over-represented in protein-rich nutrients. Our results provide baseline information for understanding the roles of dietary animal proteins in reshaping the gut microbiome. Furthermore, growth-promotion by specific species/genus may be used as a cultivation tool for uncultured gut microorganisms.

## Introduction

Meats (beef, pork and chicken) are considered to be an important part of human and animal diets due to the provision of high-quality protein, as well as fatty acids, amino acids and minerals (iron, zinc, selenium and vitamin B12) [1]. Milk is also known to be rich in amino acids, proteins, minerals and vitamins, as well as being good for muscle development [2]. Although meat and milk are comprised of different nutritional components, they are both used as sources of protein [3]. As such, the digestion of protein is essential for the growth and survival of living organisms. In fact, nearly 100% of animal proteins require some type of intestinal absorption process. Since proteins are difficult to digest, the gut microbiota is needed to help facilitate their digestion (Biffi A, 2019, Patents). Certain microbiota are used as probiotics to facilitate the digestion of amino acids in individuals who cannot easily digest protein, such as the elderly or children. For example, *Lactobacillus* is a genus of amino acid-fermenting bacteria whose members include species that utilize proteins to produce bioactive compounds, contributing to their immunoenhancing and antitumor properties [4-6].

The gut microbiota is increasingly becoming recognized for its involvement in digestion [7]. Gut microbiota can be modulated by dietary habitat and contributes to food digestion, immunomodulatory actions and gut homeostasis as well as gut diseases, metabolic disorders, and brain dysfunction [8-10]. In vivo studies investigating the changes in gut microbiota due to the dietary environment are actively under way [11-13]. However, in vitro studies examining the effects of beef, pork, chicken, and milk on the composition of intestinal microflora don’t exist, excluding other factors, such as effects caused by the host or biologically active compounds. In order to evaluate the relationship between gut microorganisms and dietary animal protein sources, it is important to identify bacteria that contribute to the use of proteins and amino acids contained in beef, pork, chicken, and milk.

In this study, we investigated changes in gut microbiota in response to beef, pork, chicken, and milk using next generation sequencing (NGS). Based on this, we predicted the microbiota that mainly used proteins and amino acids for fermentation and the function of gene clustering using the Cluster of Orthologous Groups (COGs) and Kyoto Encyclopedia of Genes and Genomes pathways (KEGG). Our results suggested that media containing animal protein sources (beef, pork, chicken, or milk) provide a novel prospect for the growth of microbiota able to digest proteins and amino acids originating from meat and milk.

## Materials and Methods

### Sample Preparation

Six-week-old pigs raised on a Kangwon National University experimental farm (Republic of Korea) were used in this study. Five pigs were randomly selected for fecal sample collection. Feces from 5 pigs were mixed, 15 g of which was equally distributed into three 50 ml tubes for reproducibility. Before incubation, to avoid the effects of fecal residues, fecal samples were mixed thoroughly with 50 ml 1× phosphate-buffered saline (PBS) and filtered with a 100-μm cell strainer (SPL Life Sciences, Republic of Korea). To make media, freeze-dried beef (round), pork (sirloin) and chicken (breast) were purchased from a local online shop, and skim milk was obtained from MB Cell (Republic of Korea). The experimental procedures were performed in accordance with the Guide for the Care and Use of Laboratory Animals and approved by the Institutional Animal Care and Use Committee of Kangwon National University (KIACUC, KW-190429-1).

### Incubation of Cultures

To obtain different types of culture media, 15 g each of beef, pork, chicken, and skim milk in freeze-dried powdered form was mixed with 500 ml of distilled water and sterilized at 170°C using an autoclave. Briefly, in aerobic condition, 1 ml each of fecal suspensions was distributed into 49 ml of beef, pork, chicken, or skim milk broth, and 1× PBS (control), respectively. The control group was set with the feces group affected by culture (A_Feces) and the feces group not affected by culture (B_Feces), and the beef (Beef), pork (Pork), chicken (Chicken) and skim milk (Milk) medium groups were set as the experimental group. Without processing the device that creates an anaerobic condition, the rest of the groups except the B_Feces group were cultured for 18 h at 36°C. B_Feces group was sampled immediately after filtering with PBS without culturing, while A_Feces group was sampled after culturing in PBS. The beef, pork, chicken, and skim milk cultures and feces groups (B_Feces, A_Feces) were analyzed three times each.

### Proximate Composition Analysis of Materials

Three samples of beef, pork, chicken, and skim milk powder, respectively, were prepared for proximate composition analysis to evaluate their nutritive value and the amount of protein. After bringing the samples to uniform amounts, they were analyzed for moisture, protein, fat and ash at the Institute of Animal Resources, Kangwon National University (Republic of Korea, Table 1). Moisture content was determined by drying the samples in an oven at 135°C for 2 h. Protein in the samples was determined by Kjeldahl method. The samples were digested by heating at 420°C for 1 h with 10 ml of concentrated sulfuric acid (H_2_SO_4_) in the presence of catalyst. After cooling the samples, they were analyzed by Kjeldahl Protein Analyzer. Crude fat was determined by ether extract method using a Soxhlet apparatus. Moisture-free 0.5 g samples were placed in timble filter and then fitted in a Soxtec System 1043 Extraction unit. Receiving beaker with 50 ml of diethyl ether was also fitted to the Soxtec Extraction unit. To extract the crude fat, samples were heated by the Soxtec unit at 100°C with 15 min boiling step, 40 min rinsing step, and 9 min recovery step. Then, ether was collected, and the fat in fixed volume bottle was dried at 105°C for 2 h. To determine crude ash, approximately 1 g of the sample was placed in the crucible and then burned at 600°C for 2 h in the furnace. Crude ash was cooled down in the desiccator for 30 min (Institute of Animal Resources, Kangwon National University).

### DNA Extraction and Sequencing

The DNA was extracted from before and after the incubation of feces (B_Feces, A_Feces, respectively), and the culture media in different types of media (Beef, Pork, Chicken, and Milk) using a NucleoSpin Soil Kit (Macherey- Nagel, Germany), according to the manufacturer’s instructions. This extracted genomic DNA was stored at -20°C until further analysis. The V4 region of 16S rRNA was amplified from the total extracted genomic DNA. Polymerase chain reaction (PCR) amplification was carried out using Takara EX-taq polymerase (Takara Bio, Japan) and the following universal primer sets: forward, 5'-GGACTACHVGGGTWTCTAAT-3'; and reverse, 5'- GTGCCAGCMGCCGCGGTAA-3'. The PCR program was as follows: 1 cycle of 94°C for 3 min, followed by 30 cycles of 94°C for 45 sec, 55°C for 1 min, 72°C for 1.5 min, and then 1 cycle of 72°C for 10 min, sequentially [14]. Before sequencing, the amplicons were normalized to 50 ng per sample using a Spark 10 M Multimode microplate eader (Tecan Group AG, Switzerland). The DNA libraries were constructed and sequenced by C&K Genomics for Illumina sequencing. The DNA libraries were sequenced using an Illumina MiSeq platform (Illumina Inc., USA), providing 2×250 base pair (bp) paired-ends.

### Microbial Community Analysis

The raw sequence reads were quality-trimmed and de-multiplexed using FastQC and in-house Perl script. The processed reads were analyzed using Quantitative Insight Into Microbial Ecology (QIIME software, version 1.9.1; http://qiime.org/index.html), the results of which were used to obtain bacterial community richness and diversity indices (rarefaction curves, Chao1, Shannon and phylogenetic diversity (PD)). The reads were clustered into operational taxonomic units (OTUs) by closed-reference OTU picking at a 97% level of sequence similarity to the GreenGenes 16S rRNA sequence database (http://greengenes.secondgenome.com/) (version 13-8). When sequencing, the number of reads comes out differently, but for normalization, we randomly selected 10 iterations based on each read and averaged them. The OTUs were normalized to 15,000 reads per sample with 10 iterations by single rarefaction. Beta diversity and Principal Coordinate Analysis (PCoA) were plotted based on UniFrac distances using EMPeror 3D visualization software. To predict functional and evolutionary genes from the microbiome, a biological observation matrix (BIOM) file including information of OTUs generated by QIIME was compared to the COGs, (http://www.ncbi.nlm.nih.gov/COG/), and KEGG (http://www.genome.jp/kegg/) pathways. Phylogenetic Investigation of Communities by Reconstruction of Unobserved States (PICRUSt), designed to predict metagenome functional content from genes, was used for the prediction of KEGG and COGs using normalized OTUs (http://picrust.github.io/picrust/). Principal Component Analysis (PCA) was performed at the genus level based on Mahalanobis distance and visualized using the R statistical package (ggbiplot) (R Foundation for Statistical Computing, Vienna, Austria) (version 3.5.0). The abundance of microbial taxa was expressed as a percentage of the total 16S rRNA gene sequences. To analyze the microbial taxa, one-way ANOVA was used to find the significant difference between groups. The abundance of microbial taxa at the genus level was visualized as heatmap using the R statistical package (gplots) (version 3.5.0). To predict the similar species found in different dietary animal protein media, in this study, we used given sequences (OTUs clustered into a 97% sequence identity threshold with GreenGenes database in QIIME script using the picking approach) to look up similar species in Basic Local Alignment Search Tool (BLAST) (https://blast.ncbi.nlm.nih.gov/Blast.cgi). We also predicted organism-level microbiome phenotypes based on 16S rRNA data sets and mapping files using BugBase (https://bugbase.cs.umn.edu/).

## Results

### Proximate Composition Analysis of Materials

To evaluate the value of animal protein sources as food, we compared the proximate composition analysis of the beef, pork, chicken, and milk groups by analyzing their contents (Table 1). The crude protein relative contents were the highest in chicken, followed by beef, pork and skim milk . The crude fat relative contents were the highest in the pork, followed by beef, chicken and skim milk. The crude ash relative contents were the highest in the pork, skim milk, beef and chicken. The crude water relative contents of materials were the highest in the skim milk, followed by chicken, pork and beef.

### Microbial Community and Diversity in Different Groups

**Fig. 1:**
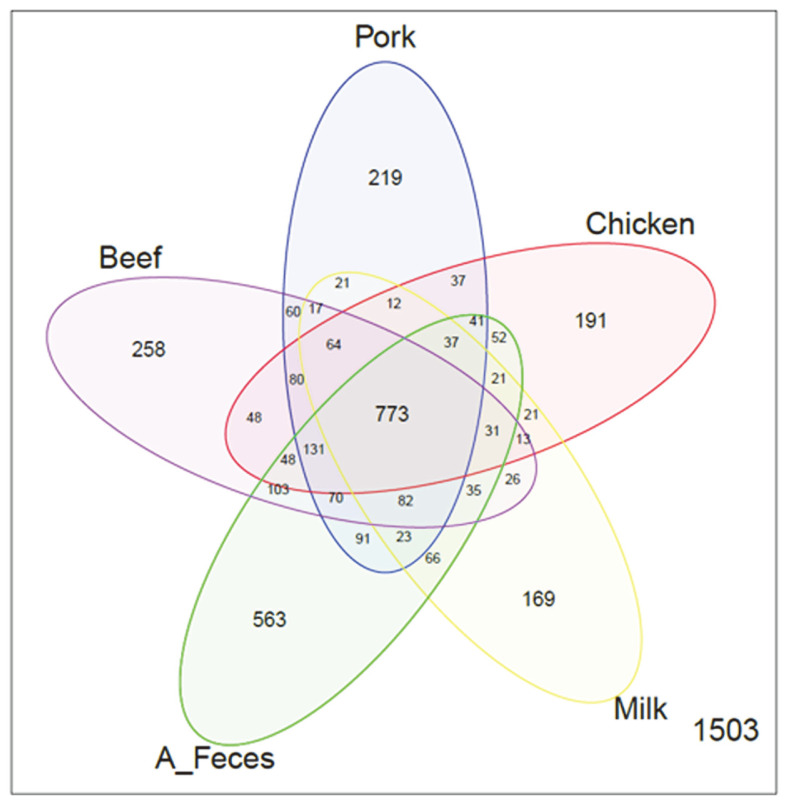
The number of the species cultured in different types of dietary animal protein media (Beef, Pork, Chicken, and Milk), compared to feces incubated (A_Feces), are shown as VennDiagram by level of OTUs.

**Fig. 2:**
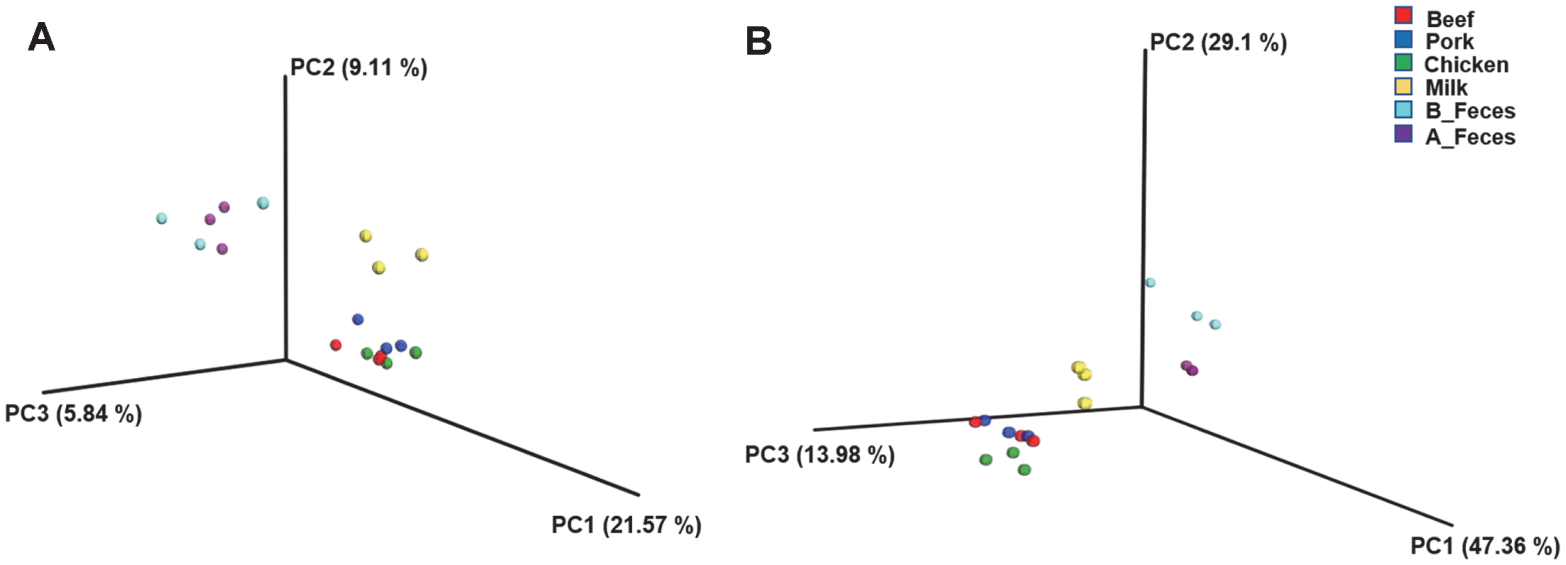
Principal coordinate analysis of unweighted (A) and weighted (B) samples based on UniFrac distances. Subject colors: red Beef (*n* = 3); blue Pork (*n* = 3); green Chicken (*n* = 3); yellow Milk (*n* = 3); mint B_Feces (*n* = 3); purple A_Feces (*n* = 3).

To compare the microbial communities and diversity among the groups, OTUs were randomly selected in a different number of reads in each sample (10, 5009, 10008, 15007, 20006, 25005, 30004, 35003, 40002, 45001, and 50000) (Fig. S1). The feces groups (A_Feces and B_Feces) showed a larger number of OTUs compared to that in the other groups (Beef, Pork, Chicken, and Milk). Among the treatment groups (Beef, Pork, Chicken and Milk), the beef group had the largest number of OTUs, while the milk groups had the least. Additionally, several diversity indices, including Shannon, Phylogenetic distance (PD), observed OTUs, and Chao1, were used to investigate bacterial diversity (Table 2). In terms of these diversity indices, the feces groups, in particular A_Feces, showed significant results compared to those of other groups as shown in the observed OTUs (Fig. S1). The microbiota of the beef, pork, chicken, milk and feces (A_Feces) groups shared 773 OTUs (Fig. 1). The numbers of unique OTUs detected were as follows: 258 for beef, 219 for pork, 191 for chicken, 169 for milk, and 563 for the feces group (A_Feces). The A_Feces groups had the largest number of unique OTUs, followed by Beef, Pork, Chicken and Milk. PcoA based on UniFrac distances was used to determine the relationship of bacteria diversity among the different samples (Fig. 2). The beef, pork, and chicken groups were clustered together, while the skim milk and feces groups (B_Feces and A_Feces) were clustered apart, both at the unweighted and the weighted level.

### Differences in Relative Abundance of Bacteria among Groups

**Fig. 3:**
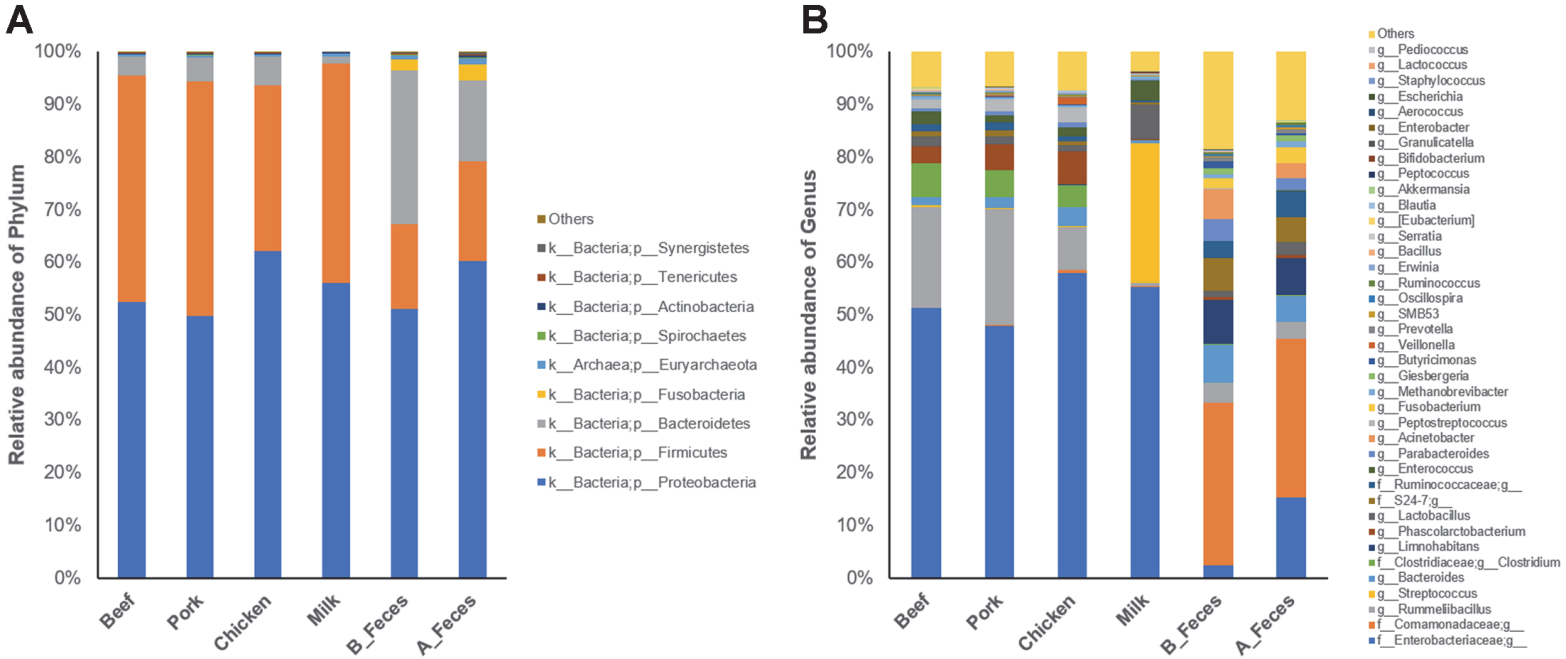
Relative abundance of bacterial community of each phylum (A) and genus (B) level in different dietary animal protein media.

The three dominant phyla detected in the all groups were Proteobacteria (52.56% in Beef, 49.88% in Pork, 62.14% in Chicken, 55.99% in Milk, 51.13% in B_Feces and 60.28% in A_Feces, respectively), Firmicutes (42.90% in Beef, 44.47% in Pork, 31.46% in Chicken, 41.74% in Milk, 16.08% in B_Feces and 18.94% in A_Feces groups, respectively), and Bacteroidetes (3.57% in Beef, 4.63% in Pork, 5.53% in Chicken, 1.44% in Milk, 29.25% in B_Feces and 15.25% in A_Feces, respectively) (Fig. 3A). After incubation, the abundance of Firmicutes significantly increased in all groups, while Bacteroidetes decreased significantly in the meat (Beef, Pork, Chicken) and milk groups.

We compared the relative abundance of the bacterial genera in the beef, pork, chicken, milk, and feces groups (Fig. 3B, Table 3) and found that some genera were represented as significantly different levels in the groups. The predominant genera in the meat groups were *Rummeliibacillus*, *Clostridium* and *Phascolarctobacterium*. In particular, *Clostridium perfringens* (OTU ID 828483) was enriched most in the meat groups (Table S1). However, three dominant genera, *Streptococcus*, *Lactobacillus* and *Enterococcus* were found in the milk group, whereas *Limnohabitans*, *Bacteroides*, and *Acinetobacter* were the predominant genera in the feces groups (Fig. 3B, Table 3).

**Fig. 4:**
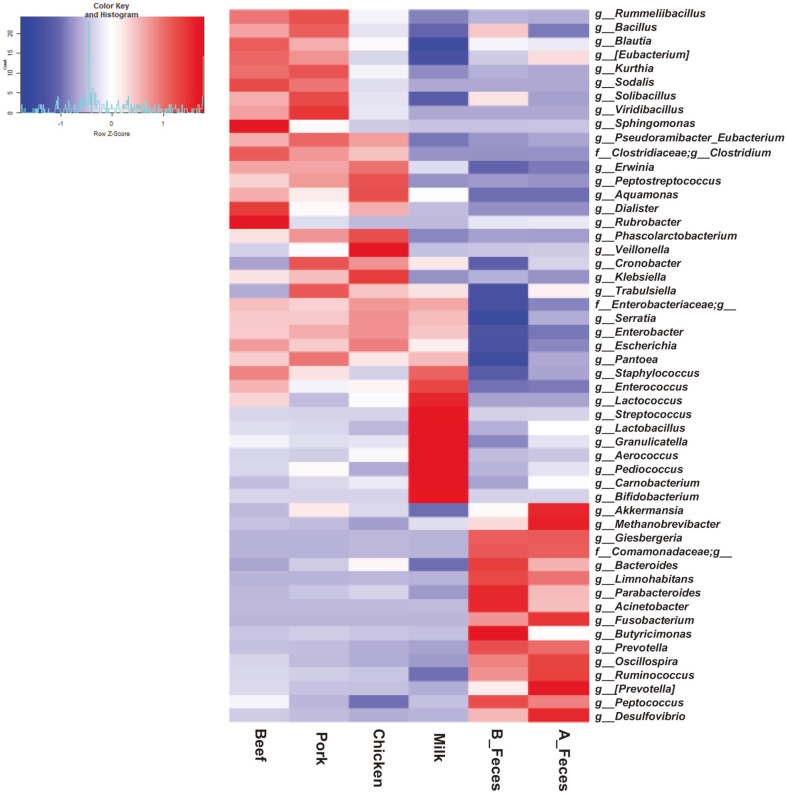
Taxonomic compositions of gut microbiota in different types of dietary animal protein media (Beef, Pork, Chicken, and Milk), and feces groups, represented as heatmap for cluster analysis of gut microbiota for 52 genera.

**Fig. 5:**
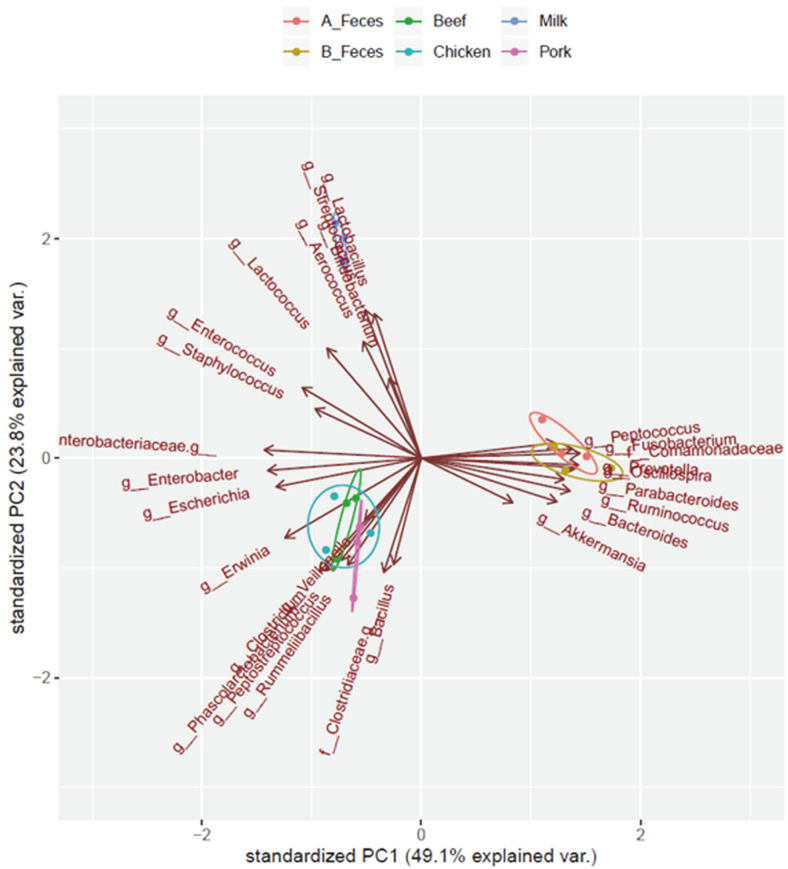
The overall composition of bacterial community in genus level using PCA. Subject colors: *green* Beef; *pink* Pork; *sky blue* Chicken; *blue* Milk; *orange* A_Feces; *gold* B_Feces.

Further characterization of the taxonomic compositions elucidated changes in the microbial compositions, with precise data (Fig. 4, Table 3). The beef, pork, and chicken groups were clustered, while the milk and feces groups were distinctly separated in their own cluster. However, certain genera were significantly more represented in each group. *Clostridium* and *Erwinia* were more significantly enriched in the meat groups (beef, pork and chicken) compared to the milk and feces groups. *Rummeliibacillus* and [*Eubacterium*] were significantly enriched only in Beef and Pork. *Blautia* and *Sphingomonas* in Beef, *Bacillus* and *Solibacillus* in Pork, and *Phascolarctobacterium* and *Veillonella* in Chicken were significantly enriched, respectively. The levels of *Serratia* and *Enterobacter* were similar in both the meat and milk groups. On the other hand, *Streptococcus, Lactobacillus,* and *Enterococcus* in the milk group were more enriched than in the meat and feces groups (B_Feces and A_Feces). *Bifidobacterium* was also more abundant in the milk group, although compared to the other groups, the difference was not significant (*P*= 0.357). The levels of *Limnohabitans, Bacteroides, Acinetobacter, Parabacteroides* and *Fusobacterium* were significantly decreased in all of the treatment groups (Beef, Pork, Chicken and Milk) but significantly higher in the feces groups. We compared the overall composition of the bacterial communities at the genus level using PCA (Fig. 5). According to the PCA plot, the microbial communities were clustered into three groups at the genus level. *Clostridium, Rummeliibacillus* and *Veillonella* in the meat groups (Beef, Pork and Chicken) were clustered together, whereas *Lactobacillus, Streptococcus,* and *Bifidobacterium* in the milk groups, and *Bacteroides, Fusobacterium* and *Ocillospira* in feces groups (B_Feces and A_Feces) were separated and clustered respectively.

### Prediction of Similar Species

Some major OTUs with the highest relative abundance were shown in Table S1, and with these OTUs, we predicted similar species. The dominant OTUs in both meat and milk groups were OTU 1111294 and OTU 4457268, sharing 97.01% identity with *Shigella* spp., and 98.59% identity with *Escherichia coli*, respectively, whereas those in feces groups were OTU 1039594 sharing 97.20% identity with *Curvibacter delicatus.* OTU 528753, OTU 828483, and OTU 252727 sharing 98.57% identity with *Phascolarctobacterium faceum*, 100% identity with *Clostridium perfringens*, and 96.35% identity with *Peptostreptococcus russellii*, respectively, were predominantly enriched in meat groups. Especially, OTU 583656, and OTU 537290 sharing 98.9% identity with *Bacteroides nordii*, and 98.08% with *Morganella morganii*, respectively, were higher in chicken group. Also, OTU 324786 sharing 97.19% identity with *Veillonella creceti / ratti* were significantly higher in chicken group. OTU 349024, and OTU 1076969 sharing 95.68% identity with *Streptococcus gallolyticus*, and 97.59% identity with *Streptococcus agalactiae,* respectively, were predominant species within *Streptococcus* genus notably enriched in milk group. OTU 288521, OTU 703741, and OTU 604966, which share 97.12% identity with *Lactobacillus equigenerosi*, 96.41% identity with *Lactobacillus acidophilus*, and 100% identity with *Lactobacillus diolivorans*, respectively, were predominant in *Lactobacillus* group found to be mostly enriched in milk group. OTU 1111582 sharing 95.99% identity with *Enterococcus faecalis* were also significantly enriched in milk group (Table S2).

### Functional Prediction in Fecal Cultures

We observed the functional pathways among the treatment groups in COGs and level 3 of KEGG pathway classes. In COGs, the meat groups were found to have a significantly higher value than the milk group in all the pathways. The ‘Amino acid transport and metabolism’, ‘Energy production and conversion’ and ‘Inorganic ion transport and metabolism’ pathways were significantly higher in the meat groups than in the milk group. Especially, ‘Transcription’, ‘Replication, combination and repair’ and ‘Cell wall/membrane/envelope biogenesis’ pathways were significantly higher in chicken group than in other groups (Table 4).

In KEGG, the meat and milk groups showed significant differences in most pathways and in metabolism, respectively (Table 5). The pathways associated with amino acid metabolism and protein processes such as ‘Arginine and proline metabolism’, ‘Glycine, serine and threonine metabolism’, ‘Butanoate metabolism’, and ‘Propanoate metabolism’ were significantly higher in meat groups. Especially, ‘Peptidases, ‘Chaperones and folding catalysts’ and ‘Cysteine and methionine metabolism’ were significantly higher in chicken group. ‘Phosphotransferase system (PTS)’, ‘Galactose metabolism’ and ‘Glycosyltransferases’ were significantly higher in the milk group.

In addition, we determined high-level phenotypes (oxygen demand, gram-positive or -negative bacteria, biofilm formation, containing mobile genetic components, oxidative stress resistance and potential genetic mobile elements and those able to tolerate stress. In addition, potentially pathogenic phenotypes were predicted in the meat and milk groups.

**Fig. 6:**
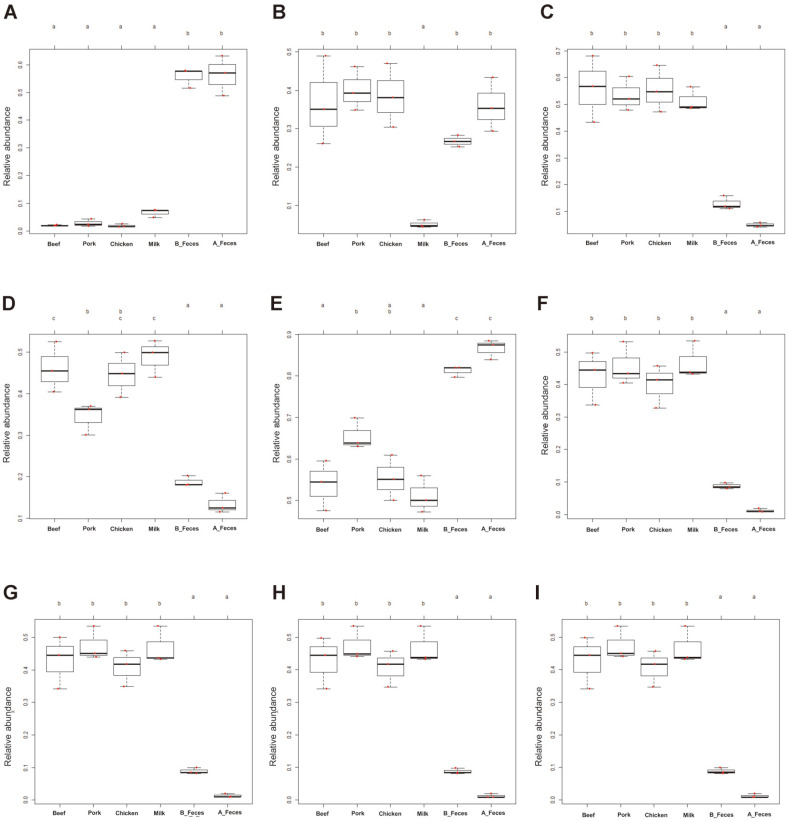
Predicted phenotypes of the bacterial community in different dietary animal proteins. (**A**) Aerobic; (**B**) Anaerobic; (**C**) Facultatively anaerobic; (**D**) Gram positive; (**E**) Gram negative; (**F**) Forms biofilms; (**G**) Contains mobile elements; (**H**) Stress tolerant; (**I**) Potentially pathogenic; Red dot means each sample. Letters (a, b, c) indicate that difference is significant at 0.05 level.

In addition, we determined high-level phenotypes (oxygen demand, gram-positive or -negative bacteria, biofilm formation, containing mobile genetic components, oxidative stress resistance and potential pathogenicity) present in microbiome samples using BugBase software program (Fig. 6). While both aerobic and anaerobic bacteria were found to be significantly higher, facultative anaerobic bacteria were lower in the feces groups (B_Feces, A_Feces). Anaerobic bacteria were significantly enriched in the meat groups (Beef, Pork and Chicken), whereas facultative anaerobic bacteria were higher in the milk group. Gram-positive bacteria were significantly enriched in beef, chicken and milk groups, while gram-negative bacteria were significantly higher in the feces groups (B_Feces, A_Feces). In Pork, both gram-positive and -negative bacteria were predicted at similar levels. The meat and milk groups were enriched in bacteria with the ability to form biofilms, those containing genetic mobile elements and those able to tolerate stress. In addition, potentially pathogenic phenotypes were predicted in the meat and milk groups.

## Discussion

In this study, we observed the effects of dietary animal proteins (beef, pork, chicken and milk) on the composition of gut microbiota for the first time in vitro using 16 S rRNA sequencing. The composition of the microbiome was dependent on different types of dietary animal proteins. Here, the levels of richness indicators (Shannon, PD, Observed OTUs and chao1) were found to be significantly higher in the feces groups (B_Feces and A_Feces), followed by the beef, pork, chicken, and milk groups. Although culturing increased the amounts of some microorganisms and demonstrated a similar pattern to feces before incubation, we assumed that limited levels of nutrition reduced the microbial diversity [15]. Although the microorganisms were in an undernourished condition, several groups of microorganisms were found to be increased. The main sugar found in milk is lactose [16]. To utilize this sugar, bacteria must express the lac operon, which encodes key enzymes such as β- galactosidase involved in the utilization and metabolism of lactose. However, it is expressed only in the presence of lactose. In some ways, using lactose is more energy intensive and less efficient than using glucose [17]. Previous studies observed that cells do not enter starvation mode in the presence of amino acids, even when nutrients are insufficient to support full survival. In addition, in the milk group, the presence of amino acids must be preceded to express β-galactosidase, which is expressed to degrade lactose by the lac operon [18-19]. Based on these findings, we suggested that the diversity of microorganisms is higher in the meat group without lactose than in the milk group with a large amount of lactose. Some products such as lactic acids, hydrogen peroxide and bacteriocin from lactic acid bacteria in milk group inhibit some bacterial growth and may reduce microbial diversity [20]. Therefore, it could be assumed that the diversity of microbiota in the meat groups would be higher than in the milk group. Furthermore, the number of unique OTUs was significantly higher in the feces groups, followed by the beef, pork, chicken, and milk groups, consistent with the alpha diversity data. This demonstrated that these animal protein sources caused certain unique OTUs to grow for each protein source. Our results suggest that these animal protein media may be used to grow specific bacteria; however, they will need to be studied and improved by further research.

In this study, we observed that the feces groups, meat groups, and milk group showed different patterns at phylum level. These results correlate with those of a previous in vivo study, wherein the consumption of meat (beef and pork) decreased the Bacteroidetes population, but increased the level of Firmicutes and Proteobacteria [11]. The consumption of chicken was also found to increase the level of Firmicutes, but decrease the Bacteroidetes population, compared to those of other protein groups [13]. Although the proportions of the major phyla in the pork group were very different from those in found in mice, the composition of the gut microbiota showed similar patterns both in vivo and in vitro. However, it was difficult to determine whether the cause of changes in the gut microbiome was due to alternations made by the host or the diet. Despite this, our results demonstrate that the effect of diet (meat and milk) may be a major contributor to alterations of the gut microbiota.

A commonly enriched genus in dietary protein (beef, pork, chicken and skim milk groups) was Enterobacteriaceae (unclassified genus). Most of Enterobacteriaceae are widely recognized as opportunistic bacteria [21]. Enterobacteriaceae are generally found in meat and dairy products [22]. The result indicated that Enterobacteriaceae did not increase significantly in A_Feces group, suggesting that the cause of the enrichment in Enterobacteriaceae could be meat and milk proteins and lactose. However, further research is needed in order to reveal the correlation between microbial and protein metabolism.

At the genus level, we observed that the genera *Clostridium, Phascolactobacterium,* and *Peptostreptococcus* were significantly predominant in the meat groups (Beef, Pork, and Chicken). The other notably enriched bacteria only in the chicken group was *Veillonella*. The *Clostridium, Peptostreptococcus* and *Veillonella* of these are known as major amino acid-fermenting bacteria, which are found along the digestive tract of humans and animals [23]. However, with exception of *V. creceti*, *Veillonella* spp. are unable to ferment amino acid, and they ferment lactate, pyruvate, malate, fumarate and oxaloacetate as a source of carbon and energy [24]. Chicken breast is considered to be homogenous white muscle in which end products such as lactate are accumulated during anaerobic respiration [25, 26]. Thus, some *Veillonella* spp. (OTU 585419, OTU 324786) may use amino acids or lactate as a source of energy, so they were enriched in chicken group this study. Some *Clostridium* spp. are gut bacteria involved in the fermentation of proteins and amino acids, such as protein-rich meat [11]. The production of short-chain fatty acids mostly uses dietary fiber and resistant starch as substrates. However, some dietary proteins can also be used as substrates for short-chain fatty acid (SCFA) products [27]. *Clostridium perfringens* use amino acid degradation as a source of energy via Stickland reaction to produce branched-chain fatty acids and ammonia [23]. In the meat media in this study, *C. perfringens* (OTU 828483) were found to play an important role in protein fermentation using proteins and amino acids from beef, pork and chicken. Furthermore, *C. perfringens* may be essential for meat protein utilization in the animal gut. However, some *C. perfringens* also secrete endotoxins, and are therefore normally recognized as pathogenic bacteria [11]. *Phascolactobacterium faceum* ferments succinate to produce propionic acids, thus they grow around succinate-producing bacteria such as *Escherichia coli* [28, 29]. Hence, it could be inferred that *Phascolactobacterium* spp. grew well in the meat where *Escherichia coli* also grew well. *Peptostreptococcus russeilli*, close to *Clostridium* phylogenetically, metabolizes amino acids to some end-products, but weakly ferments carbohydrate [30]. As such, we could nominate these bacterial species as candidates to help protein absorption, but at the same time, some of these bacterial species are known as pathogenic bacteria.

*Rummeliibacillus* spp. were the most predominant genera in the meat media, particularly in pork and beef.* Rummeliibacillus* spp., which were originally isolated from the surface of a spacecraft, are a thermophilic bacteria and physiologically recalcitrant microorganism [31]. Also, *Rummeliibacillus* are found in soil [32]. Although few studies have investigated *Rummeliibacillus* spp., they have potential applications in biomineralization [33]. A previous study isolated *Rummeliibacillus stebeskisii* from tilapia fish and found that it is used as a probiotic for improvements in growth performance, immunity, disease resistance, and gut microbiota. In addition, *R. stebeskissi* contained a proteinase for assisting in proteolysis, and this enzyme improves digestion of proteins [34, 35]. As such, we speculated that *Rummeliibacillus* spp. found in pig feces and enriched in the red meat media could have proteinase, representing a potential functional role in protein utilization. Although *Rummeliibacillus* spp. found in this study need further investigation for classification, our results indicate that *Rummeliibacillus* spp. could be used as new amino acid-fermenting bacteria and help absorption of proteins.

In the milk group, *Streptococcus, Lactobacillus,* and *Enterococcus* were significantly enriched. Some species of these bacteria produce lactic acid as the main product of sugar degradation [6, 36, 37]. These lactic acid bacteria, which are considered to be acid-tolerant bacteria, may provide a specific environment to survive by themselves, so they are often dominant at the end of milk fermentation [38]. *Streptococcus* spp. such as *S. thermophilus* and *Lactobacillus* are also involved in the hydrolysis of milk protein for initiating growth [37], and some have been recognized as amino acid-fermenting bacteria in the food industry [5]. They carry out proteolysis using casein, a protein found in milk [5, 6, 39]. Some strains of *S. agalactiae* (OTU 1076969) degrade casein and whey proteins, which is important in the dairy industry (economically important protein in milk) [40]. The advantages of *Streptococcus* spp. and *Lactobacillus* spp. are the utilization of milk proteins and lactose. The caseins and lactose in milk are the only factors that enrich the *Streptococcus* spp. and *Lactobacillus* spp. However, the majority of *Streptococcus* spp. such as *S. gallolyticus* (OTU 349024) and *S. agalactiae* (OTU 1076969) are pathogenic bacteria [41]. As such, certain *Streptococcus* spp. may help the digestion of milk proteins. *Enterococcus faecalis* is a pathogenic bacterium that also resists many kinds of stresses. However, it can also be used in the development of cheese aroma in the dairy industry [42]. Although some species or strains are known to be pathogenic bacteria, some of these microorganisms could be used to help milk protein digestion and need further investigation to be improved as bacteria used for this purpose.

In COGs, we observed that the amino acid and protein-related pathways were generally high in all the experimental groups, but the meat and milk groups had different tendencies. In particular, it was observed that the chicken group was high in some pathways. These pathways are essential for the survival and growth of microorganisms. For most functional pathways, it was unclear why their levels were higher in the chicken group. As such, further research will be needed to determine the relationship between amino acids and functional categories. In KEGG, each group showed a different functional pathway. The meat groups showed a similar pattern, with a different pattern in the milk group. This could be due to the difference in the nutritional content of meat and milk [43]. In KEGG, we observed that most amino acid metabolism-related pathways were significantly higher in the meat groups compared to those in the milk group. Especially, it was found that some amino acid metabolism-related pathways and protein processing-related pathways were significantly higher in the chicken group. Amino acid metabolism and protein processing are essential for the growth and survival of microorganisms. Furthermore, even under insufficient nutrition, microorganisms do not enter the starvation mode in the presence of amino acids [18]. On the other hand, many pathogenic bacteria such as *shigella* rely on the protein processing related pathways such as ‘Chaperones and folding catalysts’ and ‘Protein folding and associated processing’ to process virulence determinants [44]. In the chicken group, those pathways were significantly higher, suggesting that chicken protein increased bacteria involved in the process of proteins for both survival and virulence effects. Propanoate and butanoate are by-products of some amino acids in several bacteria [45]. These major functional categories suggested that the meat proteins may increase bacteria mainly involved in animal protein utilization for either growth and survival of bacteria or virulence determinants. In the milk group, the pathways involved in the synthesis, degradation and transport of sugars were significantly higher. The microorganisms found in this group may have developed mechanisms for using lactose. The use of lactose is a major function of lactic acid bacteria, and lactose hydrolysis is closely related to lactose transport. PTS is known to be involved in the transport of lactose [46]. However, further research will be needed to determine the precise differences in terms of the metabolism between the different protein groups.

In the A_Feces and B_Feces groups, the levels of oxygen demand were similar, which suggested that the changes in the bacterial community were only due to differences in protein sources (beef, pork, chicken, and milk) (Figs. 6A-6C). In all the phenotypes, it was shown that the experimental groups were higher than the control groups (Figs. 6F-6H). Biofilms, which are formed by most bacteria, occur when bacteria become attached to nutrient-rich sites (colonization) or in cases of environmental stress and stress resistance to antibiotics [47-49]. The microbiota in the protein groups studied may have developed biofilms from protein sources (beef, pork, chicken, and milk). The biofilm may contain exopolysaccharides produced by the bacteria themselves and provides an optimal environment for exchanging genetic material between cells [50]. Mobile genetic components are important factors for bacterial characterization and stabilization of indigenous species [51]. These protein sources may limit bacterial diversity and enrich specific bacteria that may use protein sources and provide advantageous features for survival. However, the formation of biofilms, mobile genetic factors, stress tolerance, and potentially pathogenic phenotypes are also associated with bacterial etiology [52, 53]. Our findings suggest that the bacteria using animal protein sources may have an advantage in survival and evolution, but this may characterize pathogenic bacteria.

The aim of our study was to understand the effects of dietary animal protein sources on the gut microbiota by observing bacterial growth in the presence of specific animal protein sources and water. Our results demonstrated that different dietary animal protein sources can be used to enrich specific bacterial species. Furthermore, our findings provide a basis for the development of a culture medium for use in the enrichment of specific bacterial species. The methods used in this study were more economical than those used in previous studies, since only water and powdered proteins were needed. In addition, our methodology facilitated mass analysis. In summary, our findings provide an insight into the role of different types of protein sources on changes in the microbiome.

## Supplemental Materials



Supplementary data for this paper are available on-line only at http://jmb.or.kr.

## Figures and Tables

**Table 1:** Proximate composition for the powdered beef, pork, chicken and skim milk.

	Beef	Pork	Chicken	Skim milk
Crude protein (%)	80.94 ± 0.17	76.84 ± 0.59	88.61 ± 0.55	33.82 ± 0.06
Crude fat (%)	13.77 ± 0.32	18.21 ± 0.30	5.26 ± 0.24	0.07 ± 0.01
Crude ash (%)	6.96 ± 0.35	11.04 ± 0.42	4.31 ± 0.27	7.80 ± 0.03
Water contents (%)	1.44 ± 0.05	1.65 ± 0.08	3.20 ± 0.02	4.89 ± 0.39

The data were written as the mean values ± standard deviation (SD).

**Table 2:** Differences in fecal microbial diversity of the different dietary animal proteins.

	Beef	Pork	Chicken	Milk	B_Feces	A_Feces	*p* value
Alpha diversity							
Shannon	3.68 ± 0.47^a^	3.68 ± 0.36^a^	3.54 ± 0.34^a^	2.99 ± 0.33^a^	5.18 ± 0.34^b^	5.19 ± 0.14^b^	<0.001***
PD	55.41 ± 3.8^bc^	53.23 ± 4.33^ab^	51.95 ± 4.88^ab^	40.95 ± 5.72^a^	62.92 ± 5.16^bc^	67.11 ± 2.72^c^	<0.001***
Observed	816.27	795.83	768.63	768.63	971.8	971.8	<0.001***
OTUs	± 85.09^ab^	± 87.06^ab^	± 85.06^ab^	± 85.06^ab^	± 95.14^bc^	± 95.14^bc^	
Chao1	1271.62	1255.86	1205.31	941.75	1489.19	1673.83	0.001**
	± 101.72^ac^	± 149.76^ab^	± 168.52^ab^	± 153.49^a^	± 168.34^bc^	± 140.87^c^	

The data were written as the mean values ± standard deviation (SD).

The *p* values were calculated using Anova test (^*^p < 0.05; ^**^p < 0.01; ^***^p < 0.001).

^a,b,c^Represents different superscripts differed significantly (*p* < 0.05).

The alpha diversity indices were calculated from 50,000 sequence reads with 10 iterations.

**Table 3:** Relative abundance of major genera in different types of dietary protein media.

Taxon	Beef	Pork	Chicken	Milk	B_Feces	A_Feces	*p* value
**Meat (Beef, Pork, and Chicken)**							
*g__Rummeliibacillus*	19.15 ± 5.07^b^	22.09 ± 4^b^	8.3 ± 2.84^a^	0.55 ± 0.36^a^	3.7 ± 0.72^a^	3.22 ± 1.55^a^	<0.001***
*g__Clostridium*	6.4 ± 2.18^b^	5.06 ± 1.76^b^	4.22 ± 0.78^b^	0.1 ± 0.03^a^	0.1 ± 0.01^a^	0.13 ± 0.04^a^	<0.001***
*g__Phascolarctobacterium*	3.35 ± 1.19^b^	4.81 ± 0.56^bc^	6.27 ± 1.9^c^	0.13 ± 0.07^a^	0.55 ± 0.16^a^	0.54 ± 0.31^a^	<0.001***
*g__Peptostreptococcus*	1.69 ± 2.00	2.2 ± 2.03	2.88 ± 1.47	0.01 ± 0.01	0.07 ± 0.08	0.02 ± 0.02	0.063
*g__Veillonella*	0.07 ± 0.03^a^	0.28 ± 0.10^a^	1.46 ± 0.96^b^	0 ± 0^a^	0.02 ± 0.00^a^	0.05 ± 0.02^a^	0.004**
*g__Erwinia*	0.26 ± 0.07^b^	0.26 ± 0.07^b^	0.31 ± 0.03^b^	0.13 ± 0^a^	0.01 ± 0.01^a^	0.04 ± 0^a^	<0.001***
*g__Bacillus*	0.22 ± 0.06^bc^	0.28 ± 0.04^c^	0.11 ± 0.04^ab^	0.01 ± 0^a^	0.19 ± 0.12^bc^	0.02 ± 0^a^	<0.001***
*g__Serratia*	0.12 ± 0.01^c^	0.12 ± 0.02^c^	0.14 ± 0.01^c^	0.12 ± 0.01^c^	0.01 ± 0.01^a^	0.06 ± 0.02^b^	<0.001***
*g__Blautia*	0.13 ± 0.02^c^	0.1 ± 0.01^bc^	0.08 ± 0.01^b^	0.02 ± 0.01^a^	0.07 ± 0.02^b^	0.07 ± 0.01^b^	<0.001***
*g__[Eubacterium]*	0.11 ± 0.03^b^	0.11 ± 0.03^b^	0.07 ± 0.02^ab^	0.03 ± 0.01^a^	0.07 ± 0.01^ab^	0.09 ± 0.03^ab^	0.009**
*g__Solibacillus*	0.09 ± 0.03^bc^	0.13 ± 0.02^c^	0.05 ± 0.01^ab^	0 ± 0^a^	0.07 ± 0.02^b^	0.03 ± 0^a^	<0.001***
*g__Sphingomonas*	0.11 ± 0.06^b^	0.03 ± 0.05^a^	0 ± 0^a^	0 ± 0^a^	0 ± 0^a^	0 ± 0^a^	0.004**
*g__Pseudoramibacter_*	0.04 ± 0.02^ab^	0.05 ± 0.03^b^	0.04 ± 0.02^ab^	0 ± 0^a^	0 ± 0.01^ab^	0.01 ± 0^ab^	0.016*
Eubacterium							
*g_Enterobacter*	0.03 ± 0^b^	0.04 ± 0.01^b^	0.04 ± 0.01^b^	0.04 ± 0^b^	0 ± 0^a^	0.01 ± 0^a^	<0.001***
*g__Pantoea*	0.01 ± 0.01^b^	0.01 ± 0^b^	0.01 ± 0.01^ab^	0.01 ± 0^b^	0 ± 0^a^	0 ± 0^ab^	0.006**
*g__Kurthia*	0.01 ± 0^b^	0.01 ± 0^b^	0 ± 0^ab^	0 ± 0^a^	0 ± 0^a^	0 ± 0^a^	0.001**
**Milk**							
*g__Streptococcus*	0.32 ± 0.03^a^	0.16 ± 0.03^a^	0.12 ± 0.04^a^	26.63 ± 1.4^b^	0.07 ± 0.01^a^	0.09 ± 0.02^a^	<0.001***
*g__Lactobacillus*	1.79 ± 0.28^a^	1.67 ± 0.77^a^	1.16 ± 0.14^a^	6.6 ± 1.28^b^	1.04 ± 0.69^a^	2.49 ± 1.73^a^	<0.001***
*g__Enterococcus*	2.49 ± 0.76^bc^	1.39 ± 0.35^ab^	1.76 ± 0.53^ac^	3.73 ± 1.75^c^	0.05 ± 0.01^a^	0.1 ± 0.06^a^	0.001**
*g__Bifidobacterium*	0 ± 0.01	0 ± 0	0 ± 0.01	0.2 ± 0.31	0 ± 0	0 ± 0	0.352
*g__Granulicatella*	0.03 ± 0.01^ab^	0.02 ± 0.01^ab^	0.03 ± 0.01^ab^	0.07 ± 0.03^b^	0.01 ± 0.01^a^	0.03 ± 0.04^ab^	0.048*
*g__Aerococcus*	0.01 ± 0^a^	0 ± 0^a^	0.01 ± 0^ab^	0.04 ± 0.03^b^	0 ± 0^a^	0 ± 0^a^	0.024*
**Feces (B_Feces, A_Feces)**							
*g__Limnohabitans*	0.01 ± 0.01^a^	0.02 ± 0.02^a^	0.15 ± 0.12^a^	0.03 ± 0.03^a^	8.44 ± 0.93^c^	7.02 ± 0.06^b^	<0.001***
*g__Bacteroides*	1.45 ± 0.86^ab^	2.16 ± 0.73^ab^	3.61 ± 0.69^ac^	0.53 ± 0.23^a^	7.3 ± 2.95^c^	4.96 ± 0.59^bc^	0.001**
*g__Acinetobacter*	0.03 ± 0.01^a^	0.03 ± 0.02^a^	0.04 ± 0.01^a^	0.07 ± 0.06^a^	5.75 ± 1.98^b^	2.75 ± 2.48^ab^	0.001**
*g__Parabacteroides*	0.54 ± 0.33^ab^	0.72 ± 0.36^ab^	0.86 ± 0.2^ab^	0.22 ± 0.1^a^	4.12 ± 1.76^c^	2.33 ± 0.17^bc^	<0.001***
*g__Fusobacterium*	0.01 ± 0^a^	0 ± 0^a^	0.01 ± 0^a^	0 ± 0^a^	2 ± 0.37^b^	3.08 ± 0.47^c^	<0.001***
*g__Giesbergeria*	0 ± 0^a^	0 ± 0^a^	0.03 ± 0.02^a^	0.01 ± 0.02^a^	1.13 ± 0.17^b^	1.14 ± 0.19^b^	<0.001***
*g__Methanobrevibacter*	0.4 ± 0.3^a^	0.38 ± 0.14^a^	0.31 ± 0.08^a^	0.47 ± 0.28^a^	0.67 ± 0.22^ab^	1.14 ± 0.15^b^	0.004**
*g__Prevotella*	0.11 ± 0.04^a^	0.11 ± 0.03^a^	0.07 ± 0.03^a^	0.05 ± 0.02^a^	0.7 ± 0.29^b^	0.63 ± 0.08^b^	<0.001***
*g__Oscillospira*	0.15 ± 0.07^a^	0.11 ± 0.03^a^	0.08 ± 0.03^a^	0.05 ± 0.03^a^	0.43 ± 0.08^b^	0.54 ± 0.04^b^	<0.001***
*g__Ruminococcus*	0.15 ± 0.05^a^	0.14 ± 0.02^a^	0.13 ± 0.02^a^	0.04 ± 0.01^a^	0.34 ± 0.04^b^	0.44 ± 0.08^b^	<0.001***
*g__[Prevotella]*	0.05 ± 0.03^a^	0.03 ± 0^a^	0.03 ± 0^a^	0.02 ± 0.01^a^	0.09 ± 0.02^a^	0.23 ± 0.08^b^	<0.001***
*g__Desulfovibrio*	0.03 ± 0.01^a^	0.02 ± 0^a^	0.01 ± 0.01^a^	0.02 ± 0.01^a^	0.08 ± 0.01^b^	0.13 ± 0.02^c^	<0.001***
*g__Peptococcus*	0.04 ± 0.02^ac^	0.03 ± 0.01^ab^	0.02 ± 0.01^a^	0.03 ± 0.02^ac^	0.07 ± 0.01^c^	0.06 ± 0.02^bc^	0.006**

The data were expressed as the mean values ± standard deviation (SD).

The *p* values were determined using Anova test (^*^p < 0.05; ^**^p < 0.01; ^***^p < 0.001).

^a,b,c^Represents different superscripts differed significantly (*p* < 0.05).

The major genera were arranged in order of abundance at each dietary protein medium

**Table 4:** Different functions predicted by PICRUSt at COGs

	Beef	Pork	Chicken	Milk	*p* value
Cellular processes and signaling	5816471±377999.1^b^	5697353±259371.6^b^	6173365±138974.3^b^	4987345±138344.3^a^	0.003**
Information storage and processing	5193771±463645.52	5076513±308720.14	5399127±153327.7	4658921±49672.14	0.071
Metabolism	10045582±721585.9^b^	9875222±487898.8^ab^	10622152±275996.1^b^	8710010±170597.4^a^	0.006**
Poorly characterized	5005496±362545.03^ab^	4939278±250441.92^ab^	5225184±168885.03^b^	4401399±63489.51^a^	0.016*
[A] RNA processing and modification	4277±179.87^a^	4019.67±213.86^a^	5120.33±217.05^b^	4490.67±503.54^ab^	0.013*
[B] Chromatin structure and dynamics	3209.67±1025.41^b^	3501.67±611.56^b^	3451.67±812.08^b^	990.67±570.37^a^	0.011*
[C] Energy production and conversion	1573935±111497.12^b^	1541481±71427.43^b^	1698296±33767.46^b^	1278554±48222.99^a^	0.001**
[D] Cell cycle control, cell division, chromosome partitioning	282634±24318.56	278584±16812.26	290374.3±8680.4	249449±2200.72	0.051
[E] Amino acid transport and metabolism	2298251±164040.8^b^	2271382±107055.38^b^	2419748±62106.42^b^	1972583±45477.79^a^	0.005**
[F] Nucleotide transport and metabolism	735090.3±69040.23	721781.7±45682.14	764439±25512.18	703316.3±6263.95	0.421
[G] Carbohydrate transport and metabolism	1880176±172511.83	1802427±115067.09	1942672±49190.07	1807801±9347.56	0.381
[H] Coenzyme transport and metabolism	1090466±95304.73^b^	1083788±67973.13^b^	1175842±38472.76^b^	875439±23775^a^	0.002**
[I] Lipid transport and metabolism	670796±21024.87^b^	667287±12323.31^b^	694460.3±31448.6^b^	561150±12810.06^a^	<0.001***
[J] Translation, ribosomal structure and biogenesis	1478325±149160.01	1463169±100286.51	1537753±61554.38	1395761±19171.54	0.397
[K] Transcription	2050886±191931.41^ab^	1993281±128859.08^ab^	2110586±82127.59^b^	1732777±27739.02^a^	0.025*
[L] Replication, recombination and repair	1657073±122212.95^ab^	1612542±80904.41^ab^	1742216±20764.12^b^	1524902±24689.92^a^	0.042*
[M] Cell wall/membrane/envelope biogenesis	1503120±130001.72^ab^	1485715±85399.44^ab^	1627713±38921.33^b^	1314289±22132.34^a^	0.010*
[N] Cell motility	657310.7±22538.48^ab^	630634.7±22261.57^ab^	705109.3±26530.78^b^	585546.7±45032.27^a^	0.008**
[O] Post-translational modification, protein turnover, and chaperones	866454±48445.47^b^	854311.7±34067.4^ab^	920225.3±16695.2^b^	773750±20498.05^a^	0.004**
[P] Inorganic ion transport and metabolism	1397135±93362.38^b^	1393335±70483.23^b^	1509541±46792.47^b^	1178174±25856.18^a^	0.002**
[Q] Secondary metabolites biosynthesis, transport, and catabolism	399731±1118.3^b^	393741±2627.54^b^	417153.3±19790.56^b^	332993±13300.19^a^	<0.001***
[R] General function prediction only	2872449±236004.18^b^	2842871±164029.64^ab^	2989845±103796.87^b^	2442005±37582.94^a^	0.012*
[S] Function unknown	2133048±126610.64^ab^	2096407±86750.13^ab^	2235339±65814.12^b^	1959394±26102.66^a^	0.024*
[T] Signal transduction mechanisms	1214394.7±114305.34^b^	1184120.3±75932.46^b^	1227335.3±68151.53^b^	886463.7±23094.22^a^	0.002**
[U] Intracellular trafficking, secretion, and vesicular transport	784593±7130.97^a^	763960.7±16969.4^a^	881245.3±17699.9^b^	757107.3±43086.33^a^	0.001**
[V] Defense mechanisms	503321.3±81694.33	495820.3±59047.98	516351.3±34766.26	416290.7±9829.84	0.173
[W] Extracellular structures	3748.67±229.1	3508.33±296.56	4196±408.02	4034±373.49	0.134
[Z] Cytoskeleton	894.33±346.15	698±241.95	814.67±358.43	415±239.06	0.299

The data were written as the mean values ± standard deviation (SD).

The *p* values were calculated using Anova test (^*^p < 0.05; ^**^p < 0.01; ^***^p < 0.001).

^a,b,c^Represent different superscripts differed significantly (*p* < 0.05).

The numeric values represented the number of reads.

**Table 5:** Different functions predicted by PICRUSt at KEGG pathways.

	Beef	Pork	Chicken	Milk	*p* value
**Meats**					
L3| Peptidases	461101±51469.4^ab^	453822.3±33693.64^ab^	484600.3±18847.4^b^	391984.7±4352.68^a^	0.039*
L3| Chaperones and folding catalysts	262772.7±15063.27^ab^	259010.3±10923.31^a^	285973.3±3992.06^b^	244529±6525.95^a^	0.007**
L3| Arginine and proline metabolism	272837.7±26601.33^b^	270659.7±17704.21^b^	285712±11222.56^b^	219739.7±4236.54^a^	0.007**
L3| Alanine, aspartate and glutamate metabolism	244570.3±25415.35	242762±18413.89	263338±9437.47	217717±2751.7	0.055
L3| Cysteine and methionine metabolism	244486.3±23890.95^ab^	243808±15940.03^ab^	252722.3±10569.91^b^	207594.3±2061.64^a^	0.028*
L3| Butanoate metabolism	225829.3±9878.33^bc^	221631.3±4595.12^b^	242377.7±6520.83^c^	200230.7±6175.87^a^	0.001**
L3| Nitrogen metabolism	218032.3±11219.03^a^	213517.3±9522.98^a^	242670±2644.82^b^	200851.3±8253.45^a^	0.002**
L3| Glycine, serine and threonine metabolism	215015.7±10023.59^b^	212849.3±6634.7^b^	229298.7±3091.94^b^	193748±5162.47^a^	0.001**
L3| Protein folding and associated processing	188203.3±5588.51^a^	183694±3826.64^a^	206154.7±700.1^b^	180022.3±7254.39^a^	0.001**
L3| Propanoate metabolism	184378±3567.89^b^	182305±2015.04^b^	192303.7±8309.38^b^	162146±4556.93^a^	0.001**
L3| Phenylalanine, tyrosine and tryptophan biosynthesis	179122.3±15575.07^b^	180056±10350.22^b^	194999±6852.53^b^	146393±4210.07^a^	0.002**
L3| Histidine metabolism	151072±16471.77^ab^	154068.7±12033.96^b^	157433.3±7721.44^b^	123570±1463.39^a^	0.019*
L3| Tyrosine metabolism	128079.3±5981.87^ab^	125931.7±3990.25^ab^	134586±3046.69^b^	121367.3±2722.41^a^	0.026*
L3| Valine, leucine and isoleucine degradation	110183.7±4639.57^b^	111560.3±3620.94^b^	111071.7±10229.86^b^	81362±3761.38^a^	0.001**
L3| Tryptophan metabolism	93888.33±3893.41^b^	94453.33±2276.63^b^	98280±5671.62^b^	73021±4146.61^a^	<0.001***
L3| beta-Alanine metabolism	80542±1326.78^bc^	80141±324.26^b^	88693.67±4197.45^c^	61366.67±4519.91^a^	<0.001***
L3| Sulfur metabolism	76189.67±2720.15^a^	74965.67±1768.28^a^	82703.67±1594.33^b^	72572.67±1717.7^a^	0.001**
L3| Lysine degradation	77949.33±3740.93^b^	77703.67±2591.12^b^	82726.67±4807.04^b^	60045±4578.56^a^	0.001**
L3| Amino acid metabolism	72142.33±4315.53^b^	70665±2086.61^b^	67531.33±2220.84^b^	52615.33±1517.55^a^	<0.001***
L3| Phenylalanine metabolism	61022±3664.5^bc^	60762±2753.06^b^	68652.33±2732.2^c^	46289.67±2707.62^a^	<0.001***
**Milk**					
L3| Phosphotransferase system (PTS)	235535.7±24472.21^a^	209999±13454.91^a^	239291±4465.16^a^	314594.7±6724.96^b^	<0.001***
L3| Galactose metabolism	149028.3±16895.43^a^	141665.7±10891.29^a^	155938.3±3368.9^ab^	175904±2499.54^b^	0.018*
L3| Glycosyltransferases	110692±2312.67^a^	107608.7±2816.76^a^	125681±1868.89^b^	129972.3±3789.04^b^	<0.001***
L3| Ascorbate and aldarate metabolism	40845.67±2199.07^a^	39028±772.49^a^	42658±1519.79^a^	46752.33±652.78^b^	0.001**
L3| D-Alanine metabolism	33050±2584.57^ab^	31977.33±1699.8^a^	35002.33±1006.08^ab^	36857.33±317.42^b^	0.027*
L3| Transcription related proteins	6060.67±239.35^ab^	5468.67±235.37^a^	6408±275.66^b^	6591±331.33^b^	0.005**
L3| Carbohydrate digestion and absorption	5644.67±418.11^a^	5503.67±285.58^a^	5757.67±522.05^a^	7188.33±358.92^b^	0.003**

The data were written as the mean values ± standard deviation (SD).

The *p* values were calculated using Anova test (^*^p < 0.05; ^**^p < 0.01; ^***^p < 0.001).

^a,b,c^Represent different superscripts differed significantly (*p* < 0.05).

The major categories were arranged in order of abundance at each dietary animal protein medium.

The numeric values represented the number of reads.
