# Supplementary material for: Changes in Gut Microbial Community of Pig Feces in Response to Different Dietary Animal Protein Media
Source: J Microbiol Biotechnol. 2020 May 31;30(9):1321–34. doi: 10.4014/jmb.2003.03021 (PMC9728240; doi:10.4014/jmb.2003.03021)
Supplement: Supplementary file 1 [file JMB-30-9-1321-supple.pdf]

J. Microbiol. Biotechnol.
https://doi.org/10.4014/jmb.2003.03021

Table **S1**. Relative abundance (%) of major OTU ID and prediction of similar species in different dietary animal protein media

| OTU ID | Taxon | Similar species | Beef | Pork | Chicken | Milk | B_Feces | A_Feces | P.value |
|---|---|---|---|---|---|---|---|---|---|
| 1111294 | f__Enterobacteriaceae; g__; s__ | *Shigella sp.* | 46.88±4.16$^{c}$ | 44.31±4.62$^{c}$ | 53.51±5.26$^{c}$ | 51.18±4.76$^{c}$ | 2.12±1.24$^{a}$ | 14.06±2.59$^{b}$ | <0.001*** |
| 1039594 | f__Comamonadaceae; g__; s__ | *Curvibacter delicatus* | 0.02±0.01$^{a}$ | 0.07±0.07$^{a}$ | 0.58±0.44$^{a}$ | 0.12±0.15$^{a}$ | 29.93±4.57$^{b}$ | 29.03±2.75$^{b}$ | <0.001*** |
| 349024 | *g__Streptococcus* | *Streptococcus gallolyticus* | 0.23±0.03$^{a}$ | 0.11±0.03$^{a}$ | 0.07±0.03$^{a}$ | 22.69±1.04$^{b}$ | 0.04±0.01$^{a}$ | 0.04±0.01$^{a}$ | <0.001*** |
| 528753 | *g__Phascolarctobacterium* | *Phascolarctobacterium faceum* | 2.74±1.01$^{b}$ | 3.99±0.57$^{bc}$ | 5.35±1.65$^{c}$ | 0.04±0.02$^{a}$ | 0.27±0.1$^{a}$ | 0.19±0.11$^{a}$ | <0.001*** |
| 828483 | *g__Clostridium* | *Clostridium perfirngens* | 4.24±1.84$^{b}$ | 3.8±1.35$^{b}$ | 3.72±0.52$^{b}$ | 0.09±0.03$^{a}$ | 0.03±0.01$^{a}$ | 0.04±0.02$^{a}$ | <0.001*** |
| 4457268 | f__Enterobacteriaceae; g__; s__ | *Escherichia coli* | 2.8±0.15$^{c}$ | 2.05±0.15$^{b}$ | 2.53±0.14$^{bc}$ | 2.37±0.48$^{bc}$ | 0.08±0.02$^{a}$ | 0.59±0.2$^{a}$ | <0.001*** |
| 1111582 | *g__Enterococcus* | *Enterococcus faecalis* | 2.25±0.64$^{bc}$ | 1.31±0.32$^{ab}$ | 1.7±0.51$^{ac}$ | 3.23±1.44$^{c}$ | 0.04±0.01$^{a}$ | 0.09±0.06$^{a}$ | <0.001*** |
| 583656 | *g__Bacteroides* | *Bacteroides nordii* | 0.92±0.62$^{ab}$ | 1.47±0.57$^{ab}$ | 2.56±0.47$^{b}$ | 0.25±0.15$^{a}$ | 1.76±0.95$^{ab}$ | 2.02±0.83$^{ab}$ | 0.013* |
| 252727 | *g__Peptostreptococcus* | *Peptostreptococcus russellii* | 1.68±2 | 2.19±2.03 | 2.87±1.48 | 0.01±0.01 | 0.07±0.08 | 0.02±0.02 | 0.065 |
| 288521 | *g__Lactobacillus* | *Lactobacillus equigenerosi* | 0.62±0.27 | 0.6±0.34 | 0.43±0.15 | 0.85±0.07 | 0.6±0.44 | 1.1±0.89 | 0.545 |
| 589277 | *g__Bacteroides* | *Bacteroides dorei* | 0.15±0.09 | 0.19±0.05 | 0.16±0.01 | 0.09±0.03 | 2.58±2.41 | 1.01±0.16 | 0.055 |
| 1076969 | *g__Streptococcus* | *Streptococcus agalactiae* | 0.03±0.01$^{a}$ | 0.01±0$^{a}$ | 0±0$^{a}$ | 2.42±1.05$^{b}$ | 0±0$^{a}$ | 0±0$^{a}$ | <0.001*** |
| 537290 | *g__Morganella* | *Morganella morganii* | 0.19±0.3 | 0.97±1.43 | 2.37±2.16 | 0.02±0.03 | 0.04±0.04 | 0.13±0.22 | 0.115 |
| 572889 | *g__Fusobacterium* | *Fusobacterium mortiferum* | 0.01±0$^{a}$ | 0±0$^{a}$ | 0±0$^{a}$ | 0±0$^{a}$ | 1.35±0.53$^{b}$ | 2.25±0.55$^{c}$ | <0.001*** |
| 851323 | *g__Parabacteroides* | *Parabacteroides johnsonii* | 0.2±0.12$^{a}$ | 0.29±0.12$^{ab}$ | 0.33±0.07$^{ab}$ | 0.07±0.02$^{a}$ | 1.38±0.41$^{c}$ | 0.77±0.12$^{b}$ | <0.001*** |
| 703741 | *g__Lactobacillus* | *Lactobacillus acidophilus* | 0.29±0.09$^{a}$ | 0.32±0.24$^{a}$ | 0.19±0.05$^{a}$ | 1.05±0.51$^{b}$ | 0.13±0.06$^{a}$ | 0.36±0.18$^{a}$ | 0.006** |
| 604966 | *g__Lactobacillus* | *Lactobacillus diolivorans* | 0.34±0.08 | 0.33±0.14 | 0.23±0.04 | 0.67±0.12 | 0.13±0.13 | 0.51±0.51 | 0.132 |
| 585419 | *g_Veillonella* | *uclassified* | 0.02±0.01$^{a}$ | 0.09±0.06$^{ab}$ | 0.69±0.54$^{b}$ | 0±0$^{a}$ | 0.01±0$^{a}$ | 0.03±0.01$^{a}$ | 0.015* |
| 324786 | *g_Veillonella* | *Veillonella creceti / ratti* | 0.03±0.01$^{a}$ | 0.12±0.08$^{ab}$ | 0.22±0.08$^{b}$ | 0±0$^{a}$ | 0±0$^{a}$ | 0±0$^{a}$ | <0.001*** |

The data were expressed as the mean values±standard deviation. (SD)

The P.values were determined using Anova test. (*P < 0.05; **P < 0.01; ***P < 0.001)

$^{a, b, c}$ represents different superscripts differed significantly. (P < 0.05)

The major OTUs were arranged in order of abundance at each dietary animal protein medium.

**Table S2**. Sequences of OTUs and identities (%) with similar species

| OTU ID | Taxon | Similar species | Similarity (%) | Sequence |
|---|---|---|---|---|
| 1111294 | f__Enterobacteriaceae; g__; s__ | *Shigella sp.* | 97.01 | AGAGTTTGATCCTGGCTCAGATTGAACGCTGGCGGCAGGCCTAACACATGCAA<br>GTCGAACGGTAACAGGAAACAGATTGCTGTTTCGCTGACGAGTGGCGGACGG<br>GTGAGTAATGTCTGGGAAACTGCCTGATGGAGGGGGATAACTACTGGAAACG<br>GTAGCTAATACCGCATAACGTCGCAAGACCAAAGAGGGGGACCCTCGGGCCT<br>CTTGCCATCGGATGTGCCCAGATGGGATTAGCTTGTTGGTGGGGTAACGGCTC<br>AACACACAGGGGATCCGATCCCTAAGCTTGTTCTGAGAGGACTGACCAGCCA<br>CACGTGGAACTGAGACACGGTTCAGACTTCTACGGGAGGCAGCAGTGGGGAA<br>TATTGCACAATGGGCGCAAGGCTGATGCAGGCATGCCGCGTGTATGAAGAAG<br>GCCTTCGGGTTGTAAAGTACTTTCAGCAGGGAGGAAGGGAGTAAAGTTAATA<br>CCTTTGCTCATTGACGTTACCCGCCAGAAGAAGCACCGGCTAACTCCGTGCCA<br>GCAGCCGCGGTAATACGGAGGGTGCAAGCGTTAATCGGAATTACTGGGCGTA<br>AAGCGCACGCAGGCGGTTTGTTAAGTCAGATGTGAAATCCCCGGGCTCAACCT<br>GGGAACTGCATCTGATACTGGCAAGCTTGAGTCTCGTAGAGGGGGGTAGAAT<br>TCCAGGTGTAGCGGTGAAATGCGTAGAGATCTGGAGGAATACCGGTGGCGAA<br>GGCGGCCCCCTGGACGAAGACTGACGCTCAGGTGCGAAAGCGTGGGGAGCAA<br>ACAGGATTAGATACCCTGGTAGTCCACGCCGTAAACGATGTCGACTTGGAGGT<br>TGTGCCCTTGAGGCGTGGCTTCCGGAGCTAACGCGTTAAGTCGACCGCCTGGA<br>GGAGTACGGCCGCAAGGTTAAAACTTCTAATGAATTGACCGGGGGCCCGCAC<br>AAGCGGTGGAGCATGTGCGTTTAATTCGATGCAACGCGAAGAATGCTTACCTG<br>GTCTTGACATTCCACGGACGTTTTCGGAGATGTGAATGTGCCTTCGGGAACCG<br>TGAAGACAGGTAGCGTGCATGGCTGTCGTCACTGCTCGTGTTGTGAAATGTTG<br>GGTTCAGTCCCGCAACGAGCTGCTATACCTCAGTGCTATACGCTCTTGTTGCC<br>AGCGGTCCGGCCGGGAACTCAAAGGAGACTGCCAGTGATAAACTGGAGGAAG<br>GTGGGGATGACGTCAAGTCATCATGGCCCTTACGACCAGGGCTACACACGTGC<br>TACAATGGCGCATACAAAGAGAAGCGACCTCGCGAGAGCAAGCGGACCTCAT<br>AAAGTGCGTCGTAGTCCGGATTGGAGTCTGCAACTCGACTCCATGAAGTCGGA<br>ATCGCTAGTAATCGTGGATCAGAATGCCACGGTGAATACGTTCCCGGGCCTTG<br>TACACACCGCCCGTCACACCATGGGAGTGGGTTGCAAAAGAAGTAGGTAGCT<br>TAACCTTCGGGAGGGCGCTTACCACTTTGTGATTCATGACTGGGGTGAAGTCG<br>TAACAAGGTAGCCGT |
| 1039594 | f__Comamonadaceae; g__; s__ | *Curvibacter delicatus* | 97.2 | ATTGAACGCTGGCGGCATGCCTTACACATGCAAGTCGAACGGCAGCACGGGA<br>GCAATCCTGGTGGCGAGTGGCGAACGGGTGAGTAATACATCGGAACGTGCCC<br>GATCGTGGGGGATAACTACTCGAAAGAGTAGCTAATACCGCATACGATCTAC<br>GGATGAAAGCGGGGGATCGCAAGACCTCGCGCGGACGGAGCGGCCGATGGC<br>AGATTAGGTAGTTGGTGGGATAAAAGCTTACCAAGCCGACGATCTGTAGCTG<br>GTCTGAGAGGACGACCAGCCACACTGGGACTGAGACACGGCCCAGACTCCTA<br>CGGGAGGCAGCAGTGGGGAATTTTGGACAATGGGCGAAAGCCTGATACAGCA<br>ATGCCGCGTGCAGGATGAAGGCCTTCGGGTTGTAAACTGCTTTTGTACGGAAC<br>GAAAAGACTCTCTCTAATAAAGGGGGTCCATGACGGTACCGTAAGAATAAGC<br>ACCGGCTAACTACGTGCCAGCAGCCGCGGTAATACGTAGGGTGCAAGCGTTA<br>ATCGGAATTACTGGGCGTAAAGCGTGCGCAGGCGGTTATGTAAGACAGAGGT<br>GAAATCCCCGGGCTCAACCTGGGAACTGCCTTTGTGACTGCATAGCTAGAGTA<br>CGGTAGAGGGGGATGGAATTCCGCGTGTAGCAGTGAAATGCGTAGATATGCG<br>GAGGAACACCGATGGCGAAGGCAATCCCCTGGACCTGTACTGACGCTCATGC<br>ACGAAAGCGTGGGGAGCAAACAGGATTAGATACCCTGGTAGTCCACGCCCTA<br>AACGATGTCAACTGGTTGTTGGGTCTTAACTGACTCAGTAACGAAGCTAACGC<br>GTGAAGTTGACCGCCTGGGGAGTACGGCCGCAAGGTTGAAACTCAAAGGAAT<br>TGACGGGGACCCGCACAAGCGGTGGATGATGTGGTTTAATTCGATGCAACGC<br>GAAAAACCTTACCCACCTTTGACATGTACGGAGGTTTCCAGAGATGGATTCGT<br>GCTCGAAAGAGAACCGTAACACAGGTGCTGCATGGCTGTCGTCAGCTCGTGTC<br>GTGAGATGTTGGGTTAAGTCCCACAACGAGCGCAACCCTTGCCATTAGTTGCT<br>ACATTTAGTTGGGCACTCTAATGGGACTGCCGGTGACAAACCGGAGGAAGGT<br>GGGGATGACGTCAAGTCCTCATGGCCCTTATAGGTGGGGCTACACACGTCATA<br>CAATGGCTGGTACAAAGGGTTGCCAACCCGCGAGGGGGAGCTAATCCCATAA<br>AGCCAGTCGTAGTCCGGATCGCAGTCTGCAACTCGACTGCGTGAAGTCGGAAT<br>CGCTAGTAATCGTGGATCAGCATGTCACGGTGAATACGTTCCCGGGTCT |

| | | | | |
|---|---|---|---|---|
| 349024 | *g__Streptococcus* | *Streptococcus gallolyticus* | 95.68 | AGAGTTTGATCCTGGCTCAGGATGAACGCTGGCGGCGTGCCTAATACATGCAA<br>GTCGAACGCACAGCGAAAGGTGCTTGCACCTTTCAAGTGAGTGGCGAACGGG<br>TGAGTAACACGTGGATAACCTACCTCAAGGTTGGGGATAACATTTGGAAACA<br>GATGCTAATACCGAATAAAACTTCGTATCGCATGATACAAGGTTAAAAGGCG<br>CTACGGCGTCACCTAGAGATGGATCCGCGTTGTATTAGCTAGTTGGTGGGGTA<br>ACGGCCTACCAAGGCGACGATACATAGCCGACCTGAGAGGGTGATCGGCCAC<br>ACTGGGACTGAGACACGGCCCAGACTCCTACGGGAGGCAGCAGTAGGGAATC<br>TTCGGCAATGGGGGCAACCCTGACCGAGCAACGCCGCGTGAGTGAAGAAGGT<br>TTTCGGATCGTAAAGCTCTGTTGTAAGAGAAGAACGTGTATGAGAGTGGAAA<br>GTTCACACAGTGACGGTAACTTACCAGAAAGGGACGGCTAACTACGTGCCAG<br>CAGCCGCGGTAATACGTAGGTCCCGAGCGTTGTCCGGATTTATTGGGCGTAAA<br>GCGAGCGCAGGCGGTTTAATAAGTCTGAAGTTAAAGGCAGTGGCTTAACCATT<br>GTTCGCTTTGGAAACTGTTAAACTTGAGTGCAGAAGGGGAGAGTGGAATTCCA<br>TGTGTAGCGGTGAAATGCGTAGATATATGGAGGAACACCGGTGGCGAAAGCG<br>GCTCTCTGGTCTGTAACTGACGCTGAGGCTCGAAAGCGTGGGGAGCAAACAG<br>GATTAGATACCCTGGTAGTCCACGCCGTAAACGATGAGTGCTAGGTGTTAGGC<br>CCTTTCCGGGGCTTAGTGCCGCAGCTAACGCATTAAGCACTCCGCCTGGGGAG<br>TACGACCGCAAGGTTGAAACTCAAAGGAATTGACGGGGGCCCGCACAAGCGG<br>TGGAGCATGTGGTTTAATTCGAAGCAACGCGAAGAACCTTACCAGGTCTTGAC<br>ATCCCGATGCTATTTCTAGAGATAGAAAGTTTCTTCGGAACATCGGTGACAGG<br>TGGTGCATGGTTGTCGTCAGCTCGTGTCGTGAGATGTTGGGTTAAGTCCCGCA<br>ACGAGCGCAACCCCTATTGTTAGTTGCCATCATTGAGTTGGGCACTCTAGCGA<br>GACTGCCGGTAATAAACCGGAGGAAGGTGGGGATGACGTCAAATCATCATGC<br>CCATTATGACCTGGGCTACACACGTGCTACAATGGTTGGTACAACGAGTCGCA<br>AGTCGGTGACGGCAAGCAAATCTCTTAAAGCCAATCTCAGTCCGGATTGTAGG<br>CTGCAACTCGCCTACATGAAGTCGGAATCGCTAGTAATCGCGGATCAGCACGC<br>CGCGGTGAATACGTTCCCGGGCCTTGCACTCACCGCCCGTCA |
| 528753 | *g__Phascolarctobacterium* | *Phascolarctobacterium faceum* | 98.57 | GAGTTTGATCCTGGCTCAGGACGAACGCTGGCGGCGTGCCTAACACATGCAA<br>GTCGAACGGAGAATTTTATTTCGGTAGGATTCTTAGTGGCGAACGGGTGAGTA<br>ACGCGTAGGCAACCTGCCCTTTAGACGGGACAACATTCCGAAAGGAGTGCTA<br>ATACCGGATGAGATCATTTTCCCGCATAGGAGAGTGAAGAAAGATGGCCTCT<br>ACATGTAAGCTATCGCTAAAGGATGGGCCTGCGTCTGATTAGCTAGTTGGTAG<br>TGTAACGGACTACCAAGGCGATGATCAGTAGCCGGTCTGAGAGGATGAACGG<br>CCACATTGGGACTGAGACACGGCCCAAACTCCTACGGGAGGCAGCAGTGGGG<br>AATCTTCCGCAATGGACGAAAGTCTGACGGAGCAACGCCGCGTGAGTGATGA<br>AGGATTTCGGTCTGTAAAACTCTGTTGTTTATGACGAACGTGCAGTGTGTGAA<br>CAATGCATTGCAATGACGGTAGTAAACGAGGAAGCCACGGCTAACTACGTGC<br>CAGCAGCCGCGGTAATACGTAGGTGGCGAGCGTCGTCCGGAATTATTGGGCG<br>TAAAGAGCATGTAGGCGGCCTATTAAGTCGAGCGTGAAAATGCGGGGCTCAA<br>CCCCGTATGGCGCTGGAAACTGGTAGGCTTGAGTGCAGGAGAGGAAAGGGGA<br>ATTCCCAGTGTAGCGGTGAAATGCGTAGATATTGGGAGGAACACCAGTGGCG<br>AAGGCGCCTTTCTGGACTGTGTCTGACGCTGAGATGCGAAAGCCAGGGTAGC<br>GAACGGGATTAGATACCCCGGTAGTCCTGGCCGTAAACGATGGGTACTAGGT<br>GTAGGAGGTATCGACCCCTTCTGTGCCGGAGTTAACGCAATAAGTACCCCGCC<br>TGGGGAGTACGGCCGCAAGGTTGAAACTCAAAGGAATTGACGGGGGCCCGCA<br>CAAGCGGTGGAGTATGTGGTTTAATTCGACGCAACGCGAAGAACCTTACCAA<br>GGCTTGACATTGATTGATCGCTCTAGAGATAGAGCTTTCCCTTCGGGGACAAG<br>AAAACAGGTGGTGCATGGCTGTCGTCAGCTCGTGTCGTGAGATGTTGGGTTAA<br>GTCCCGCAACGAGCGCAACCCCTATCCTATGTTACCAGCAAGTGAAGTTGGGG<br>ACTCATGGGAGACTGCCAGGGACAACCTGGAGGAAGGCGGGGATGACGTCAA<br>GTCATCATGCCCCTTATGTCTTGGGCTACACACGTACTACAATGGTCGGAAAC<br>AGAGGGAAGCAAAGCCGCGAGGCAGAGCAAACCCCAGAAACCCGATCTCAG<br>TTCGGATCGCAGGCTGCAACTCGCCTGCGTGAAGTCGGAATCGCTAGTAATCG<br>CAGGTCAGCATACTGCGGTGAATACGTTCCCGGGCCTTG |

| | | | | |
|---|---|---|---|---|
| 828483 | *g__Clostridium* | *Clostridium perfirngens* | 100 | TTGATCCTGGCTCAGGATGAACGCTGGCGGCGTGCTTAACACATGCAAGTCGA<br>GCGAGAGAGTTCCTTCGGGAACAATCTAGCGGCGGACGGGTGAGTAACACGT<br>GGGCAACCTGCCTCATAGAGGGGAATAGCCTCCCGAAAGGGAGATTAATACC<br>GCATAATATCATTGAGTGGCATCATTTAATGATTAAAGGAGTAATCCGCTATG<br>AGATGGGCCCGCGGCGCATTAGCTAGTTGGTGAGGTAACGGCTCACCAAGGC<br>GACGATGCGTAGCCGACCTGAGAGGGTGATCGGCCACATTGGGACTGAGACA<br>CGGCCCAGACTCCTACGGGAGGCAGCAGTGGGGAATATTGCACAATGGGGGA<br>AACCCTGATGCAGCAACGCCGCGTGAGTGATGAAGGTCTTCGGATTGTAAAG<br>CTCTGTCTTCAGGGACGATAATGACGGTACCTGAGGAGGAAGCCACGGCTAA<br>CTACGTGCCAGCAGCCGCGGTAATACGTAGGTGGCAAGCGTTATCCGGATTTA<br>CTGGGCGTAAAGGGAGCGTAGGCGGATAATTAAGTGGGATGTGAAATACCCG<br>GGCTCAACTTGGGTGCTGCATTCCAAACTGGTTATCTAGAGTGCAGGAGAGGA<br>GAGTGGAATTCCTAGTGTAGCGGTGAAATGCGTAGAGATTAGGAAGAACACC<br>AGTGGCGAAGGCGACTCTCTGGACTGTAACTGACGCTGAGGCTCGAAAGCGT<br>GGGGAGCAAACAGGATTAGATACCCTGGTAGTCCACGCCGTAAACGATGAAT<br>ACTAGGTGTAGGGGCCCCAAGCCTCTGTGCCGCCGCTAACGCATTAAGTATTC<br>CGCCTGGGGAGTACGGTCGCAAGATTAAAACTCAAAGGAATTGACGGGGACC<br>CGCACAAGCAGCGGAGCATGTGGTTTAATTCGAAGCAACGCGAAGAACCTTA<br>CCTAGACTTGACATCTCCTGAATTACTCTTAATCGAGGAAGTCCCTTCGGGGA<br>CAGGAAGACAGGTGGTGCATGGTTGTCGTCAGCTCGTGTCGTGAGATGTTGGG<br>TTAAGTCCCGCAACGAGCGCAACCCTTATTGTTAGTTGCTACCATTAAGTTGA<br>GCACTCTAGCAAGACTGCCCGGGTTAACCGGGAGGAAGGTGGGGATGACGTC<br>AAATCATCATGCCCCTTATGTCTAGGGCTACACACGTGCTACAATGGCAAGTA<br>CAAAGAGAAGCAAGACCGCAAGGTGGAGCAAAACTCAAAAACTTGTCTCAGT<br>TCGGATTGTAGGCTGAAACTCGCCTACATGAAGCTGGAGTTGCTAGTAATCGC<br>GAATCAGCATGTCGCGGTGAATACGTTCCCGGGTCTTGTACACACCGCCCGTC<br>ACACCATGAGAGTTGGCAATACCCGAAGTCCGTGAGCTAACCGCAAGGAGGC<br>AGCGGCCGAAGGTAGGGTCAGCGATTGGGGTGAAGTCGTAACAAGGTAGCCG<br>TAGGAGAACCTGCGGCTGGATCACCTCCTT |
| 4457268 | f__Enterobacteriaceae; g__;<br>s__ | *Escherichia coli* | 98.59 | GGTAACAGGAAAGAAGCTTGCTTTCTTTGCTGACGAGTGGCGGACGGGTGAG<br>TAATGTCTGGGAAACTGCCTGATGGAGGGGGATAACTACTGGAAACGGTAGC<br>TAATACCGCATAACGTCGCAAGACCAAAGAGGGGGACCTTCGGGCCTCTTGC<br>CATCGGATGTGCCCAGATGGGATTAGCTAGTAGGTGGGGTAACGGCTCACCTA<br>GGCGACGATCCCTAGCTGGTCTGAGAGGATGACCAGCCACACTGGAACTGAG<br>ACACGGTCCAGACTCCTACGGGAGGCAGCAGTGGGGAATATTGCACAATGGG<br>CGCAAGCCTGATGCAGCCATGCCGCGTGTATGAAGAAGGCCTTCGGGTTGTAA<br>AGTACTTTCAGCGGGGAGGAAGGGAGTAAAGTTAATACCTTTGCTCATTGACG<br>TTACCCGCAGAAGAAGCACCGGCTAACTCCGTGCCAGCAGCCGCGGTAATA<br>CGGAGGGTGCAAGCGTTAATCGGAATTACTGGGCGTAAAGCGCACGCAGGCG<br>GTTTGTTAAGTCAGATGTGAAATCCCCGGGCTCAAACCTGGGAACTGCATCT<br>GATACTGGCAAGCTTGAGTCTCGTAGAGGGGGGTAGAATTCCAGGTGTAGC<br>GGTGAAATGCGTAGAGATCTGGAGGAATACCGGTGGCGAAGGCGGCCCCCTG<br>GACGAAGACTGACGCTCAGGTGCGAAAGCGTGGGGAGCAAACAGGATTAGAT<br>ACCCTGGTAGTCCACGCCGTAAACGATGTCGACTTGGAGGTTGTGCCCTTGAG<br>GCGTGGCTTCCGGAGCTAACGCGTTAAGTCGACCGCCTGGGGAGTACGGCCC<br>GCAAGGTTAAAACTCAAATGAATTGACGGGGGCCCGCACAAGCGGTGGAG<br>CATGTGGTTTAATTCGATGCAACGCGAAGAACCTTACCTGGGTCTTGACATCC<br>ACAGNAACTTTCCAGAAAATGGATTGGGTGCCTTCGGGAACTGTGAGACAGG<br>TGCTGCATGGCTGTCGTTCACCTCGTGTTGTGAAATGTTGGGTTAAGTCCCGCA<br>ACGAGCGCAACCCTTATCCTTTGTTGCCAGCGGTCCGGCCGGGAACTCAAAGG<br>AAACTGCCAGTGATAAACTGGAGGAAGGTGGGGATGACGTCAAGTCATCATG<br>GCCCTTACGACCAGGGCTACACACGTGCTACAATGGCGCATACAAAGAGAAG<br>CGACCTCGCGAGAGCAAGCGGACCTCATAAAGTGCGTCGTAGTCCGGATTGG<br>AGTCTGCAACTCGACTCCATGAAGTCGGAATCGCTAGTAATCGTGGATCAGAA<br>TGCCACGGTGAATACGTTCCCGGGCCTTGTACACACCGCCCGTCACACCATGG<br>GAGTGGGTTGCAAAAGAAGTAGGTAGGCTTAACCCTTCGGGAGGGGCGCTAC<br>CAC |

| | | | | |
|---|---|---|---|---|
| 1111582 | *g__Enterococcus* | *Enterococcus faecalis* | 95.99 | AGAGTTTGATCATGGCTCAGGATGAACGCTGGCGGCGTGCCTAATACATGCAA<br>GTCGAGCGAACAGACGAGGAGCTTGCTCCTTTGACGTTAGCGGCGGACGGGT<br>GAGTAACACGTGGATAACCTACCTATAAGACTGGGATAACTTCGGGAAACCG<br>GAGCTAATACCGGATAACATGTTGAACCGCATGGTTCAACAGTGAAAGACGG<br>TCTTGCTGTCACTTATAGATGGATCCGCGCCGCATTAGCTAGTTGGTGAGGTA<br>ACGGCTCACCAAGGCCACGATGCATAGCCGACCTGAGAGGGTGATCGGCCAC<br>ACTGGGACTGAGACACGGCCCAGACTCCTACGGGAGGCAGCAGTAGGGAATC<br>TTCGGCAATGGACGAAAGTCTGACCGAGCAACGCCGCGTGAGTGAAGAAGGT<br>TTTCGGATCGTAAAACTCTGTTGTTAGAGAAGAACAAGGACGTTAGTAACTGA<br>ACGTCCCCTGACGGTATCTAACCAGAAAGCCACGGCTAACTACGTGCCAGCA<br>GCCGCGGTAATACGTAGGTGGCAAGCGTTGTCCGGATTTATTGGGCGTAAAGC<br>GAGCGCAGGCGGTTTCTTAAGTCTGATGTGAAAGCCCCCGGCTCAACCGGGG<br>AGGGTCATTGGAAACTGGGAGACTTGAGTGCAGAAGAGGAGAGTGGAATTCC<br>ATGTGTAGCGGTGAAATGCGTAGATATATGGAGGAACACCAGTGGCGAAGGC<br>GGCTCTCTGGTCTGTAACTGACGCTGAGGCTCGAAAGCGTGGGGAGCAAACA<br>GGATTAGATACCCTGGTAGTCCACGCCGTAAACGATGAGTGCTAAGTGTTGGA<br>GGGTTTCCGCCCTTCAGTGCTGCAGCAAACGCATTAAGCACTCCGCCTGGGGA<br>GTACGACCGCAAGGTTGAAACTCAAAGGAATTGACGGGGGCCCGCACAAGCG<br>GTGGAGCATGTGGTTTAATTCGAAGCAACGCGAAGAACCTTACCAGGTCTTGA<br>CATCCTTTGACCACTCTAGAGATAGAGCTTTCCCTTCGGGGACAAAGTGACAG<br>GTGGTGCATGGTTGTCGTCAGCTCGTGTCGTGAGATGTTGGGTTAAGTCCCGC<br>AACGAGCGCAACCCTTATTGTTAGTTGCCATCATTTAGTTGGGCACTCTAGCG<br>AGACTGCCGGTGACAAACCGGAGGAAGGTGGGGATGACGTCAAATCATCATG<br>CCCCTTATGACCTGGGCTACACACGTGCTACAATGGGAAGTACAACGAGTCGC<br>TAGACCGCGAGGTCATGCAAATCTCTTAAAGCTTCTCTCAGTTCGGATTGCAG<br>GCTGCAACTCGCCTGCATGAAGCCGGAATCGCTAGTAATCGCGGATCAGCAC<br>GCCGCGGTGAATACGTTCCCG |
| 583656 | *g__Bacteroides* | *Bacteroides nordii* | 98.9 | CTGGCTCAGGATGAACGCTAGCTACAGGCTTAACACATGCAAGTCGAGGGGC<br>ATCAGGATGTAGCAATACATTGCTGCGACCGGCGCACGGGTGAGTAACACGT<br>ATCCAACCTGCCTTTTACTCGGGGATAGGCTTTCGAAAGAAAGATTAATACCC<br>GATGGTATAATATTTCCGCATGGGATTATTATTAAAGAATTTCGGTAAAAGGT<br>GGGGATGCGTTCCATTAGGCAGTTGGCGGGGTAACGGCCCACCAAACCTTCG<br>ATGGATAGGGGTTCTGAGAGGAAGGTCCCCCACATTGGAACTGAGACACGGT<br>CCAAACTCCTACGGGAGGCAGCAGTGAGGAATATTGGTCAATGGGCGAAAGC<br>CTGAACCAGCCAAGTAGCGTGAAGGATGACTGCCCTATGGGTTGTAAACTTCT<br>TTTATAGGGGAATAAAATGAGCCACGTGTGGCTTTTTGTATGTACCCTATGAA<br>TAAGGATCGGCTAACTCCGTGCCAGCAGCCGCGGTAATACGGAGGATCCGAG<br>CGTTATCCGGATTTATTGGGTTTAAAGGGAGCGTAGGTGGACATGTAAGTCAG<br>TTGTGAAAGTTTGCGGCTCAACCGTAAAATTGCAGTTGATACTGTGTGTCTTG<br>AGTACAGTAGAGGTGGGCGGAATTCGTGGTGTAGCGGTGAAATGCTTAGATA<br>TCACGAAGAACTCCGATTGCGAAGGCAGCTCACTGGACTGCAACTGACACTG<br>ATGCTCGAAAGTGTGGGTATCAAACAGGATTAGATACCCTGGTAGTCCACACA<br>GTAAACGATGAATACTCGCTGTTTGCGATATACAGTAAGCGGCCAAGCGAAA<br>GCATTAAGTATTCCACCTGGGGAGTACGCCGGCAACGGTGAAACTCAAAGGA<br>ATTGACGGGGGCCCGCACAAGCGGAGGAACATGTGGTTTAATTCGATGATAC<br>GCGAGGAACCTTACCCGGGCTTAAATTGCATTTGAATATATTGGAAACAGTAT<br>AGCCGTAAGGCAAATGTGAAGGTGCTGCATGGTTGTCGTCAGCTCGTGCCGTG<br>AGGTGTCGGCTTAGGTGCCATAACGAGCGCAGCCCTTATCTTTAGTTACTAAC<br>AGGTCATGCTGAGGACTCTAGAGAGACTGCCGTCGTAAGATGTGAGGAAGGT<br>GGGGATGACGTCAAATCAGCACGGCCCTTACGTCCGGGGCTACACACGTGTTA<br>CAATGGGGGGTACAGAAGGCAGCTACCTGGTGACAGGATGCTAATCCCAAAA<br>GCCTCTCTCAGTTCGGATCGAAGTCTGCAACCCGACTTCGTGAAGCTGGATTC<br>GCTAGTAATCGCGCATCAGCCATGGCGCGGTGAATACGTTCCCGGGCCTTGCA |

| | | | | |
|---|---|---|---|---|
| 252727 | *g__Peptostreptococcus* | *Peptostreptococcus russellii* | 96.35 | GATGAACGCTGGCGGCGTGCTTAACACATGCAAGTCGAGCGCGGTCCCTTTGG<br>TGCTTGCACCAGAGAAGACTGAGCGGCGGACGGGTGAGTAACGCGTGGGTAA<br>CCTGCCCTATACACATGGATAACATGCTGAAAAGCATACTAATACATGATAAC<br>GTACACTGATGGCATCTTCGGTGTATCAAAGCGTTAGCGGTATAGGATGGACC<br>CGCGTCTGATTAGCTAGTTGGTGGGGTAAAAGCCTACCAAGGCCACGATCAGT<br>AGCCGACCTGAGAGGGTGATCGGCCACATTGGAACTGAGACACGGTCCAAAC<br>TCCTACGGGAGGCAGCAGTGGGGAATATTGCACAATGGGCGAAAGCCTGATG<br>CAGCAACGCCGCGTGAACGATGAAGGTCTTCGGATCGTAAAGTTCTGTCGCAG<br>GGGAAGATAATGACGGTACCCTGTGAGGAAGCACCGGCTAACTACGTGCCAG<br>CAGCCGCGGTAATACGTAGGGTGCTAGCGTTATCCGGATTTACTGGGCGTAAA<br>GGGTGCGTAGGTGGTCTTTCAAGTCGGTGGTTAAAGGCTACGGCTCAACCGTA<br>TTAAGCCGCCGAAACTGGAAGACTTGAGTGCAGGAGAGGAAAGTGGAATTCT<br>CAGTGTAGCGGTGAAATGCGTAGATATTGAGAAGAACACCAGTAGCGAAGGC<br>GGCTTTCTGGACTGTAACTGACACTGAGGCACGAAAGCGTGGGTAGCAAACA<br>GGATTAGATATCCTGGTAGTCCACGCCGTAAACGATGAGTATTAGGTGTCGGG<br>GGTTACCCCCCTCGGTGCCGCAGTTAACGCATTAAATACTCCGCCTGGGGAGT<br>ACGCACGCAAGTGTGAAACTCAAAGGAATTGACGGGGACCCGCACAAGTAGC<br>GGAGCATGTGGTTTAATTCGAAGCAACGCGAAGAACCTTACCTAAGCTTGACA<br>TCTCTCAGATCGGTAGTTAATCCTACCTTCCCTTCGGGGCTGAGATGACAGGT<br>GGTGCATGGTTGTCGTCAGCTCGTGTCGTGAGATGTTGGGTTAAGTCCCGCAA<br>CGAGCGCAACCCTTGTCTTTAGTTGCCAGCATTAAGTTGGGCACTCTAGAGAG<br>ACTGCCAGGGATAACCTGGAGGAAGGTGGGGATGACGTCAAATCATCATGCC<br>CCTTATGTCTTGGGCTACACACGTACTACAATGGTCGGCAACAGAGGGAAGCA<br>AAGCCGTGAGGCAGAGCAAACCCCAGAAACCCGATCCCAGCTCGGATTGCAG<br>GCTGCAACTCGCCTGGATGAAGTCGGAATCGCTAGTAATCGCAGGTCAGCATA<br>CTGCGGTGAATACGTTCCCGGGCCTTGTACACACCGCCCGTCACACCACGAAA<br>GTTGGTAACACCCGAAGCCGGTGGGGTAACCGTAAGGAGCCAGCCGTCTAAG<br>GTGGGGCCGATGATTGGGGTG |
| 288521 | *g__Lactobacillus* | *Lactobacillus equigenerosi* | 97.12 | AGAGTTTGATCCTGGCTCAGGATGAACGCCGGCGGTGTGCCTAATACATGCAA<br>GTCGTACGCGTTGGCCCAACTGATTTGAAGTGCTTGCACGGATCTGACGTTGG<br>TTTACCAACGAGTGGCGGACGGGTGAGTAACACGTAGGTAACCTGCCCAGAA<br>GCGGGGGACAACACTTGGAAACAGGTGCTAATACCGCATAACAATTCAAATC<br>GCATGATTTGAATTTGAAAGATGGTTTCGGCTATCACTTCTGGATGGACCTGC<br>GGCGCATTAGCTTGTTGGTGGGGTAATGGCCTACCAAGGCAGTGATGCGTAGC<br>CGAGTTGAGAGACTGATCGGCCACAATGGGACTGAGATACGGCCCATACTCC<br>TACGGGAGGCAGCAGTAGGGAATCTTCCACAATGGGCGCAAGCCTGATGGAG<br>CAACACCGCGTGAGTGAAGAAGGTCTTCGGATCGTAAAGCTCTGTTGTTGAAG<br>AAGAACGTGCATGAGAGTAACTGTTCATGCAGTGACGGTATTCAACCAGAAA<br>GTCACGGCTAACTACGTGCCAGCAGCCGCGGTAATACGTAGGTGGCAAGCGT<br>TATCCGGATTTATTGGGCGTAAAGCGAGCGCAGGCGGTTGCTTAGGTCTGATG<br>TGAAAGCCTTCGGCTTAACCGAAGAAGGGCATCGGAAACCGGGCGACTTGAG<br>TGCAGAAGAGGACAGTGGAACTCCATGTGTAGCGGTGGAATGCGTAGATATA<br>TGGAAGAACACCAGTGGCGAAGGCGGCTGTCTGGTCTGCAACTGACGCTGAG<br>GCTCGAAAGCATGGGTAGCGAACAGGATTAGATACCCTGGTAGTCCATGCCG<br>TAAACGATGAGTGCTAGGTGTTGGAGGGTTTCCGCCCTTCAGTGCCGGAGCTA<br>ACGCATTAAGCACTCCGCCTGGGGAGTACGACCGCAAGGTTGAAACTCAAAG<br>GAATTGACGGGGGCCCGCACAAGCGGTGGAGCATGTGGTTTAATTCGAAGCT<br>ACGCGAAGAACCTTACCAGGTCTTGACATCTTGCGCTAACCTAAGAGATTAGG<br>CGTTCCCTTCGGGGACGCAATGACAGGTGGTGCATGGTCGTCGTCAGCTCGTG<br>TCGTGAGATGTTGGGTTAAGTCCCGCAACGAGCGCAACCCTTGTTACTAGTTG<br>CCAGCATTCAGTTGGGCACTCTAGTGAGACTGCCGGTGACAAACCGGAGGAA<br>GGTGGGGACGACGTCAGATCATCATGCCCCTTATGACCTGGGCTACACACGTG<br>CTACAATGGACGGTACAACGAGTTGCGAACTCGCGAGAGTAAGCTAATCTCTT<br>AAAGCCGTTCTCAGTTCGGACTGTAGGCTGCAACTCGCCTACACGAAGTCGGA<br>ATCGCTAGTAATCGCGGATCAGCATGCCGCGGTGAATACGTTCCCGGGCCTTG<br>CACACAACGCCCGTCA |

| | | | | |
|---|---|---|---|---|
| 589277 | *g__Bacteroides* | *Bacteroides dorei* | 98.83 | GATGAACGCTAGCTACAGGCTTAACACATGCAAGTCGAGGGGCAGCATGGTCTTAGCTTGCTAAGGCTGATGGCGACCGGCGCACGGGTGAGTAACACGTATCCAACCTGCCGTCTACTCTTGGCCAGCCTTCTGAAAGGAAGATTAATCCAGGATGGGATCATGAGTTCACATGTCCGCATGATTAAAGGTATTTTCCGGTACACGATGGGGATGCGTCCCATTTAGATAGTAGGCGGGGTAACGGCCCACCTAGTCAACGATGGATAGGGGTTCTGAGAGGAAGGTCCCCCACATTGGAACTGAGACACGGTCCAAACTCCTACGGGAGGCAGCAGTGAGGAATATTGGTCAATGGGCGATGGCCTGAACCAGCCAAGTAGCGTGAAGGATGACTGCCCTATGGGTCGTAAACTTCTTTTATAAAGGAATAAAGTCGGGTATGCATACCCGTTTGCATGTACTTTATGAATAAGGATCGGCTAACTCCGTGCCAGCAGCCGCGGTAATACGGAGGATCCGAGCGTTATCCGGATTTATTGGGTTTAAAGGGAGCGTAGATGGATGTTTAAGTCAGTTGTGAAAGTTTGCGGCTCAACCGTAAAATTGCAGTTGATACTGGATGTCTTGAGTGCAGTTGAGGCAGGCGGAATTCGTGGTGTAGCGGTGAAATGCTTAGATATCACGAAGAACTCCGATTGCGAAGGCAGCCTGCTAAGCTGCAACTGACATTGAGGCTCGAAAGTGTGGGTATCAAACAGGATTAGATACCCTGGTAGTCCACACGGTAAACGATGAATACTCGCTGTTTGCGATATACGGCAAGCGGCCAAGCGAAAGCGTTAAGTATTCCACCTGGGGAGTACGCCGGCAACGGTGAAACTCAAAGGAATAGACGGGGGCCCGCACAAGCGGAGGAACATGTGGTTTAATTCGATGATACGCGAGGAACCTTACCCGGGCGTAAATTGCACTCGAATGATCCGGAAACGGTTCAGCTAGCAATAGCGAGTGTGAAGGTGGTGCATGGTTGTCGTCAGCTCGTGCCGTGAGGTGTCGGCTTAAGTGCCATAACGAGCGCAACCCTCGTTGTCAGTTACTAACAGGTGATGCTGAGGACTCTGACAAGACTGCCATCGTAAGATGTGAGGAAGGTGGGGAGGACGTCAAATCAGCACGGCCCCTACGTCCGGGGGTACACACGTGTTACAATGGGGGGGACAGAGGGCCGCTACCACGCGAGTGGATGCCAATCCCTAAAACCCCTCTCAGTTCGGACTGGAGTGTGCAACCCGACTCCACGAAGCTGGATTCGCTAGTAATCGCGCATCAGCCACGGCGCGGTGAAAAAGATCCCGGGCCTTGTACA |
| 1076969 | *g__Streptococcus* | *Streptococcus agalactiae* | 97.59 | GACGAACGCTGGCGGCGTGCCTAATACATGCAAGTAGAACGCTGAGGTTTGGTGTTTACACTAGACTGATGAGTTGCGAACGGGTGAGTAACGCGTAGGTAACCTGCCTCATAGCGGGGGATAACTATTGGAAACGATAGCTAATACCGCATAAGAGTAATTAACACATGTTGGTTATTTAAAAGGAGCAATTGCTTCACTGTGAGATGGACCTGCGTTGTATTAGCTAGTTGGTGAGGTAAAGGCTCACCAAGGCGACGATACATAGCCGACCTGAGAGGGTGATCGGCCACACTGGGACTGAGACACGGCCCAGACTCCTACGGGAGGCAGCAGTAGGGAATCTTCGGCAATGGACGGAAGTCTGACCGAGCAACGCCGCGTGAGTGAAGAAGGTTTTCGGATCGTAAAGCTCTGTTGTTAGAGAAGAACGTTGGTAGGAGTGGAAAATCTACCAAGTGACGGTAACTAACCAGAAAGGGACGGCTAACTACGTGCCAGCAGCCGCGGTAATACGTAGGTCCCGAGCGTTGTCCGGATTTATTGGGCGTAAAGCGAGCGCAGGCGGTTCTTTAAGTCTGAAGTTAAAGGCAGTGGCTTAACCATTGTACGCTTTGGAAACTGGAGGACTTGAGTGCAGAAGGGGAGAGTGGAATTCCATGTGTAGCGGTGAAATGCGTAGATATATGGAGAAACACCGGTGGCGAAAGCGGCTCTCTGGTCTGTAACTGACGCTGAGGCTCGAAAGCGTGGGGAGCAAACAGGATTAGATACCCTGGTAGTCCACGCCGTAAACGATGAGTGCTAGGTGTTAGGCCCTTTCCGGGGCTTAGTGCCGCAGCTAACGCATTAAGCACTCCGCCTGGGGAGTACGACCGCAAGGTTGAAACTCAAAGGAATTGACGGGGGCCCGCACAAGCGGTGGAGCATGTGGTTTAATTCGAAGCAACGCGAAGAACCTTACCAGGTCTTGACATCCTTCTGACCGGCCTAGAGATAGGCTTTCTCTTCGGAGCAGAAGTGACAGGTGGTGCATGGTTGTCGTCAGCTTGTGTCGTGAGATGTTGGGTTAAGTCCCGCAACGAGCGCAACCCCTATTGTTAGTTGCCATCATTAAGTTGGGCACTCTAGCGAGACTGCCGGTAATAAACCGGAGGAAGGTGGGGATGACGTCAAATCATCATGCCCCTTATGACCTGGGCTACACACGTGCTACAATGGACAATACAAAGGGCAGCGAAACCGCGAGGTCAAGCAAATCCCATAAAGTTGTTCTCAGTTCGGATTGTAGTCTGCAACTCGACTACATGAAGCTGGAATCGCTAGTAATCGTAGATCAGCATGCTACGGTGAATACGTTCCCGGGTCT |

| | | | | |
|---|---|---|---|---|
| 537290 | *g__Morganella* | *Morganella morganii* | 98.08 | TAGAGTTTGATCCTGGCTCAGATTGAACGCTGGCGGCAGGCCTAACACATGCA<br>AGTCGGGCGGTAACAGGGAGAAGCTTGCTTCTCTGCTGACGAGCGGCGGACG<br>GGTGAGTAATGTATGGGGATCTGCGTGATGGAGGGGGATAACTCTGGAAAC<br>GGTAGCTAATACCGCATAATGTCTTCGGACCAAAGCGGGGGACCTCCGGGCCT<br>CGCGCCATCAGATGAACCCATATGGGATTAGCTTGTAGGTGAGGTAACGGCTC<br>ACCTAGGCGACGATCCCTAGCTGGTCTGAGAGGATGATCAGCCACACTGGGA<br>CTGAGACACGGCCCAGACTCCTACGGGAGGCAGCAGTGGGGAATATTGCACA<br>ATGGGCGCAAGCCTGATGCAGCCATGCCGCGTGTATGAAGAAGGCCTTCGGG<br>TTGTAAAGTACTTTCAGTCGGGAGGAAGGTGTTAAGGTTAATAACCTTAGCAA<br>TTGACGTTACCGACAGAAGAAGCACCGGCTAACTCCGTGCCAGCAGCCGCGG<br>TAATACGGAGGGTGCAAGCGTTAATCGGAATTACTGGGCGTAAAGCGCACGC<br>AGGCGGTTGATTGAGTCAGATGTGAAATCCCCGGGCTTAACCCGGGAATTGCA<br>TCTGATACTGGTCAGCTAGAGTCTTGTAGAGGGGGGTAGAATTCCATGTGTAG<br>CGGTGAAATGCGTAGAGATCTGGAGGAATACCGGTGGCGAAGGCGGCCCCCT<br>GGACAAAGACTGACGCTCAGGTGCGAAAGCGTGGGGAGCAAACAGGATTAG<br>ATACCCTGGTAGTCCACGCCGTAAACGATGTCGACTTGGAGGTTGTGCCCTTG<br>AGGCGTGGCTTCCGGAGCTAACGCGTTAAGTCGACCGCCTGGGGAGTACGGC<br>CGCAAGGTTAAAACTCAAATGAATTGACGGGGGCCCGCACAAGCGGTGGAGC<br>ATGTGGTTTAATTCGATGCAACGCGAAGAACCTTACCTACTCTTGACATCCAG<br>AGAACTTAGCAGAGATGCTTTGGTGCCTTCGGGAACTCTGAGACAGGTGCTGC<br>ATGGCTGTCGTCAGCTCGTGTTGTGAAATGTTGGGTTAAGTCCCGCAACGAGC<br>GCAACCCTTATCCTTTGTTGCCAGCGGTTCGGCCGGGAACTCAAAGGAGACTG<br>CCAGTGATAAACTGGAGGAAGGTGGGGATGACGTCAAGTCATCATGGCCCTT<br>ACGAGTAGGGCTACACACGTGCTACAATGGCATATACAAAGAGAAGCGACCT<br>CGCGAGAGCAAGCGGACCTCATAAAGTATGTCGTAGTCCGGATTGGAGTCTG<br>CAACTCGACTCCATGAAGTCGGAATCGCTAGTAATCGTGGATCAGAATGCCAC<br>GGTGAATACGTTCCCGGGCCTTGCACTCACCGCCCGTCA |
| 572889 | *g__Fusobacterium* | *Fusobacterium mortiferum* | 98.71 | GAGTTTGATCCTGGCTCAGGATGAACGCTGACAGAATGCTTAACACATGCAAG<br>TCTACTTGATCCTTCGGGTGATGGTGGCGGACGGGTGAGTAACGCGTAAAGAA<br>CTTGCCCTGCAGTCTGGGACAACATTTGGAAACGAATGCTAATACCGGATATT<br>ATGCGAGATTCGCATGTTTCTCGTATGAAAGCTATATGCGCTGCAGGAGAGCT<br>TTGCGTCCTATTAGCTAGTTGGTGAGGTAATGGCTCACCAAGGCGATGATAGG<br>TAGCCGGCCTGAGAGGGTGAACGGCCACAAGGGGACTGAGACACGGCCCTTA<br>CTTCTACGGGAGGCAGCAGTGGGGAATATTGGACAATGGACCAAAAGTCTGA<br>TCCAGCAATTCTGTGTGCACGATGAAGTTTTTCGGAATGTAAAGTGCTTTCAG<br>TTGGGACGAAGTAAGTGACGGTACCAACAGAAGAAGCGACGGCTAAATACGT<br>GCCAGCAGCCGCGGTAATACGTATGTCGCAAGCGTTATCCGGATTTATTGGGC<br>GTAAAGCGCGTCTAGGCGGTTTGGTAAGTCTGATGTGAAAATGCGGGGCTCA<br>ACTCCGTATTGCGTTGGAAACTGTCAAACTAGAGTACTGGAGAGGTGGGCGG<br>AACTACAAGTGTAGAGGTGAAATTCGTAGATATTTGTAGGAATGCCGATGGG<br>GAAGCCAGCCCACTGGACAGATACTGACGCTAAAGCGCGAAAGCGTGGGTAG<br>CAAACAGGATTAGATACCCTGGTAGTCCACGCCGTAAACGATGATTACTAGGT<br>GTTGGGGGTCGAACCTCAGCGCCCAAGCTAACGCGATAAGTAATCCGCCTGG<br>GGAGTACGTACGCAAGTATGAAACTCAAAGGAATTGACGGGGACCCGCACAA<br>GCGGTGGAGCATGTGGTTTAATTCGACGCAACGCGAGGAACCTTACCAGCGTT<br>TGACATCCTAGGAAGTTGGCAGAGATGCCTTCGTGCCCCTTCGGGGGAACCTA<br>GTGACAGGTGGTGCATGGCTGTCGTCAGCTCGTGTCGTGAGATGTTGGGTTAA<br>GTCCCGCAACGAGCGCAACCCCTTTCGTATGTTGCCATCATTAAGTTGGGAAC<br>TCATGCGATACTGCCTGCGATGAGCAGGAGGAAGGTGGGGATGACGTCAAGT<br>CATCATGCCCCTTATACGCTGGGCTACACACGTGCTACAATGGGTAGTACAGA<br>GAGTCGCTAACCTGCGAGGGGGAGCTAATCTCAGAAAACTATTCTCAGTTCGG<br>ATTGTACTCTGCAACTCGAGTACATGAAGTTGGAATCGCTAGTAATCGCAAAT<br>CAGCTATGTTGCGGTGAATACGTTCTCGGGTCTTGTACACACCGCCCGTCACA<br>CCACGAGAGTTGGTTGCACCTGAAGTAGTAGGCCTAACCGCAAGGAGGGATG<br>CTCCGAGGGTGTGATTAGCGATTGGGGTGAAGTCGTAACAAGGTAGCCGTAG |

| | | | | |
|---|---|---|---|---|
| 851323 | *g__Parabacteroides* | *Parabacteroides johnsonii* | 97.55 | CCTGGCTCAGGATGAACGCTAGCGACAGGCTTAACACATGCAAGTCGAGGGG<br>CAGCACAGGTAGCAATACCGGGTGGCGACCGGCGCACGGGTGAGTAACGCGT<br>ATGCAACTTACCTATCAGAGGGGGATAACCCGGCGAAAGTCGGACTAATACC<br>GCATGAAGCAGGGGCCCCGCATGGGGATATTTGCTAAAGATTCATCGCTGATA<br>GATAGGCATGCGTTCCATTAGGCAGTTGGCGGGGCAACGGCCCACCAAACCG<br>ACGATGGATAGGGGTTCTGAGAGGAAGGTCCCCCACATTGGTACTGAGACAC<br>GGACCAAACTCCTACGGGAGGCAGCAGTGAGGAATATTGGTCAATGGGCGTA<br>AGCCTGAACCAGCCAAGTCGCGTGAAGGATGAAGGATCTATGGTTTGTAAAC<br>TTCTTTTATAGGGGAATAAAGTGTGGGACGTGTCCCATTTTGTATGTACCCTAT<br>GAATAAGCATCGGCTAACTCCGTGCCAGCAGCCGCGGTAATACGGAGGATGC<br>GAGCGTTATCCGGATTTATTGGGTTTAAAGGGTGCGTAGGTGGTAATTTAAGT<br>CAGCGGTGAAAGTTTGTGGCTCAACCATAAAATTGCCGTTGAAACTGGGTTAC<br>TTGAGTGTGTTTGAGGTAGGCGGAATGCGTGGTGTAGCGGTGAAATGCATAG<br>ATATCACGCAGAACTCCAATTGCGAAGGCAGCTTACTAAACCATAACTGACAC<br>TGAAGCACGAAAGCGTGGGTATCAAACAGGATTAGATACCCTGGTAGTCCAC<br>GCAGTAAACGATGATTACTAGGAGTTTGCGATACACAGTAAGCTCTACAGCG<br>AAAGCGTTAAGTAATCCACCTGGGGAGTACGCCGGCAACGGTGAAACTCAAA<br>GGAATTGACGGGGGCCCGCACAAGCGGAGGAACATGTGGTTTAATTCGATGA<br>TACGCGAGGAACCTTACCCGGGTTTGAACGTAGTCAGACCGGCCTTGAAAGA<br>GGTCTTCTAGCAATAGCTGATTACGAGGTGCTGCATGGTTGTCGTCAGCTCGT<br>GCCGTGAGATGTCGGCCTAAGTGCCATAACGAGCGCAACCCTTATCACTAGTT<br>ACTAACAGGTCGTGCTGAGGACTCTGGTGAGACTGCCAGCGTAAGCTGTGAG<br>GAAGGTGGGGATGACGTCAAATCAGCACGGCCCTTACATCCGGGGCGACACA<br>CGTGTTACAATGGCATGGACAAAGGGCAGCTACCTGGCGACAGGATGCTAAT<br>CTCTAAACCATGTCTCAGTTCGGATCGGAGTCTGCAACTCGACTCCGTGAAGC<br>TGGATTCGCTAGTAATCGCGCATCAGCCATGGCGCGGTGAATACGTTCCCGGG<br>CCTTGCACACACCGCCC |
| 703741 | *g__Lactobacillus* | *Lactobacillus acidophilus* | 96.41 | TAATACATGCAAGTCGAGCGAGCTGAATTCAAAGATCCCTTCGGGGGGATTTG<br>TTGGACGCTAGCGGCGGATGGGTGAGTAACACGTGGGCAATCTGCCCTAAAG<br>ACTGGGATACCACTTGGAAACAGGTGCTAATACCGGATAACAACATGAATCG<br>CATGATTCAAGTTTGAAAGGCGGCGCAAGCTGTCACTTTAGGATGAGCCCGCG<br>GCGCATTAGCTAGTTGGTGGGGTAAAGGCCTACCAAGGCAATGATGCGTAGC<br>CGAGTTGAGAGACTGATCGGCCACATTGGGACTGAGACACGGCCCAAACTCC<br>TACGGGAGGCAGCAGTAGGGAATCTTCCACAATGGACGAAAGTCTGATGGAG<br>CAACGCCGCGTGAGTGAAGAAAGTCTTCGGATCGTAAAGCTCTGTTGTTGGTG<br>AAGAAGGATAGAGGCAGCAACTGGTCTTTATTTGACGGTAATCAACCAGAAA<br>GTCACGGCTAACTACGTGCCAGCAGCCGCGGTAATACGTAGGTGGCAAGCGT<br>TGTCCGGATTTATTGGGCGTAAAGCGAGCGCAGGCGGAAAAATAAGTCTAAT<br>GTGAAAGCCCTCGGCTTAACCGAGGAACTGCATCGGAAACTGTTTTTCTTGAG<br>TGCAGAAGAGGAGAGTGGAACTCCATGTGTAGCGGTGGAATGCGTAGATATA<br>TGGAAGAACACCAGTGGCGAAGGCGGCTCTCTGGTCTGCAACTGACGCTGAG<br>GCTCGAAAGCATGGGTAGCGAACAGGATTAGATACCCTGGTAGTCCATGCCG<br>TAAACGATGAGTGCTAAGTGTTGGGAGGTTTCCGCCTCTCAGTGCTGCAGCTA<br>ACGCATTAAGCACTCCGCCTGGGGAGTACGACCGCAAGGTTGAAACTCAAAG<br>GAATTGACGGGGGCCCGCACAAGCGGTGGAGCATGTGGTTTAATTCGAAGCA<br>ACGCGAAGAACCTTACCAGGTCTTGACATCTAGTGCAATCTGTAGAGATATGG<br>AGTTCCCTTCGGGGACGCTAAGACAGGTGGTGCATGGCTGTCGTCAGCTCGTG<br>TCGTGAGATGTTGGGTTAAGTCCCGCAACGAGCGCAACCCTTGTTATTAGTTG<br>CCAGCATTAAGTTGGGCACTCTAATGAGACTGCCGGTGACAAACCGGAGGAA<br>GGTGGGGATGACGTCAAGTCATCATGCCCCTTATGACCTGGGCTACACACGTG<br>CTACAATGGGCAGTACAACGAGAAGCAAGCCTGCGAAGGCAAGCGAATCTCT<br>GAAAGCTGTTCTCAGTTCGGACTGCAGTCTGCAACTCGACTGCACGAAGCTGG<br>AATCGCTAGTAATCGCGGATCAGCACGCCGCGGTGAATACGTTCCCGGGCCTT<br>GTACACACCGCCCGTCACACCATGGGAGTCTGCAATGCCCAAAGCCGGTGGC<br>CTAACCTTCGGGAAGGAGCCGTCTAAGGCAGGGCAGATGACTTGGGGGTGAA<br>GTCGTAACAAGGTAGCCGTAGGAGAACC |

| | | | | |
|---|---|---|---|---|
| 604966 | *g__Lactobacillus* | *Lactobacillus diolivorans* | 100 | GACGAGTGGCGAACTGGTGAGTAACACGTGGGTAACCTGCCCTTGAAGTAGA<br>GGATAACACTTGGAAACAGGTGCTAATACTGCATAACAACGAAAACCGCCTG<br>GTTTTCGTTTGAAAGATGGCTTCGGCTATCGCTTTAGGATGGACCCGCGGCGT<br>ATTAGCTAGTTGGTGAGGTAACGGCTCACCAAGGCAATGATACGTAGCCGAC<br>CTGAGAGGGTAATCGGCCACATTGGGACTGAGACACGGCCCAAACTCCTACG<br>GGAGGCAGCAGTAGGGAATCTTCCACAATGGACGAAAGTCTGATGGAGCAAC<br>GCCGCGTGAGTGATGAAGGGTTTCGGCTCGTAAAACTCTGTTGTTGGAGAAGA<br>ACGGGTGTCAGAGTAACTGTTGACATCGTGACGGTATCCAACCAGAAAGCCA<br>CGGCTAACTACGTGCCAGCAGCCGCGGTAATACGTAGGTGGCAAGCGTTGTCC<br>GGATTTATTGGGCGTAAAGCGAGCGCAGGCGGTTTTTTAGGTCTGATGTGAAA<br>GCCTTCGGCTTAACCGGAGAAGTGCATCGGAAACCGGGAGACTTGAGTGCAG<br>AAGAGGACAGTGGAACTCCATGTGTAGCGGTGAAATGCGTAGATATATGGAA<br>GAACACCAGTGGCGAAGGCGGCTGTCTGGTCTGCAACTGACGCTGAGGCTCG<br>AAAGCATGGGTAGCGAACAGGATTAGATACCCTGGTAGTCCATGCCGTAAAC<br>GATGAGTGCTAAGTGTTGGAGGGTTTCCGCCCTTCAGTGCTGCAGCTAACGCA<br>TTAAGCACTCCGCCTGGGGAGTACGACCGCAAGGTTGAAACTCAAAGGAATT<br>GACGGGGGCCCGCACAAGCGGTGGAGCATGTGGTTTAATTCGATGCTACGCG<br>AAGAACCTTACCAGGTCTTGACATCTTCTGCCAACCTAAGAGATTAGGCGTTC<br>CCTTCGGGGACAGAATGACAGGTGGTGCATGGTTGTCGTCAGCTCGTGTCGTG<br>AGATGTTGGGTTAAGTCCCGCAACGAGCGCAACCCTTATTGTTAGTTGCCAGC<br>ATTTAGTTGGGCACTCTAGCAAGACTGCCGGTGACAAACCGGAGGAAGGTGG<br>GGATGACGTCAAATCATCATGCCCCTTATGACCTGGGCTACACACGTGCTACA<br>ATGGACGGTACAACGAGTCGCGAAACCGCGAGGTCAAGCTAATCTCTTAAAG<br>CCGTTCTCAGTTCGGATTGTAGGCTGCAACTCGCCTACATGAAGTTGGAATCG<br>CTAGTAATCGTGGATCAGCATGCCACGGTGAATACGTTCCCGGGCCTTGTACA<br>CACCGCCCGTCACACCATGAGAGTTTGTAACACCCAAAGCCGGTGAGGTAAC<br>CTTCGGGGGCCAGCCGTCTAAGGTGGGACAGATGATTAGGGTGAAGTCGTAA<br>CAAGGTAGCCGTAGGAGAACCTGCGGCTGGATCACCTCCTT |
| 585419 | *g_Veillonella* | *uclassified* | - | ATGGGAGCTTGCTCCTATCATCTTAGTGGCGAACGGGTGAGTAACGCGTAATC<br>ACCTGCCCTTCAATGGGGGACAACAGTTGGAAACGACTGCTAATACCGCATAC<br>GTCGGCATCGAGAAAGGTGGCCTTTTTAAAGCTATCGTTGAAGGAGGGGATTG<br>CGTCTGATTAGCTAGTTGGAGGGGTAACGGCCCACCAAGGCAATGATCAGTA<br>GCCGGTCTGAGAGGATGAACGGCCACATTGGGACTGAGACACGGCCCAAACT<br>CCTACGGGAGGCAGCAGTGGGGAATCTTCCGCAATGGACGAAAGTCTGACGG<br>AGCAACGCCGCGTGAGTGATGAAGGTCTTCGGATTGTAAAGCTCTGTTAATCG<br>GGACGAAGAGCCTAGTGTGAATAATGCTAGGAAGTGACGGTACCGGAATAGA<br>AAGCCACGGCTAACTACGTGCCAGCAGCCGCGGTAATACGTAGGTGGCAAGC<br>GTTGTCCGGAATTATTGGGCGTAAAGCGCGCGCAGGCGGATCTGCCAGTCTGT<br>CTTAAAAGTTCGGGGCTTAACCCCGTGATGGGATGGAAACTACAGATCTAGA<br>GTATCGGAGAGGAAAGTGGAATTCCTAGTGTAGCGGTGAAATGCGTAGATAT<br>TAGGAAGAACACCAGTGGCGAAGGCGACTTTCTGGACGAAAACTGACGCTGA<br>GGCGCGAAAGCCAGGGGAGCGAACGGGATTAGATACCCCGGTAGTCCTGGCC<br>GTAAACGATGGGTACTAGGTGTAGGAGGATCGACCTTCTGTGCCGCAGTTAAC<br>GCAATAAGTACCCCGCCTGGGGAGTACGACCGCAAGGTTGAAACTCAAAGGA<br>ATTGACGGGGGCCCGCACAAGCGGTGGAGTATGTGGTTTAATTCGACGCAAC<br>GCGAAGAACCTTACCAGGTCTTGACATTGATGGACAGAACTAGAGATAGTTCC<br>TCTTCTTCGGAAGCCAGAAAACAGGTGGTGCACGGTTGTCGTCAGCTCGTGTC<br>GTGAGATGTTGGGTTAAGTCCCGCAACGAGCGCAACCCCTATCTTATGTTGCC<br>AGCACTAAGGTGGGAACTCATGAGAGACTGCCGCAGACAATGCGGAGGAAG<br>GCGGGGATGACGTCAAATCATCATGCCCCTTATGACCTGGGCTACACACGTAC<br>TACAATGGGCTTTATAAAGGGAAGCGAAGTCGCGAGGCGGAGCAAACCCCAG<br>AAACAAGCTCTCAGTTCGGATCGTAGGCTGCAACTCGCCTACGTGAAGTCGGA<br>ATCGCTAGTAATCGCAGGTCAGATACTGCGGTGAATACGTTCCCGGGCCTTGT<br>ACACACCGCCCGTCACACCACGAAAGTCGGAAGTGCCCAAAGCCGG |

| | | | | |
|---|---|---|---|---|
| 324786 | *g_Veillonella* | *Veillonella creceti / ratti* | 97.19 | AGAGTTTGATCCTGGCTCAGGACGAACGCTGGCGGCGTGCTTAACACATGCAA<br>GTCGAACGGAGAGCGATGGAAGCTTGCTTCTATCAATCTTAGTGGCGAACGG<br>GTGAGTAACGCGTAACCAACCTGCCCTTCAGAGGGGGATAACAACGGGAAAC<br>CGTTGCTAATACCGCGTACGATTAAGCTTCGGCATCGAAGCTTAATGAAAGGT<br>GGCCTCTATATATAAGCTATCGCTGAAGGAGGGGGTTGCGTCTGATTAGCTAG<br>TTGGAGGGGTAACGGCCCACCAAGGCAATGATCAGTAGCCGGTCTGAGAGGA<br>TGAACGGCCACATTGGGACTGAGACACGGCCCAAACTCCTACGGGAGGCAGC<br>AGTGGGGAATCTTCCGCAATGGACGAAAGTCTGACGGAGCAACGCCGCGTGA<br>GTGATCACGGCCTTCGGGTTGTAAAGCTCTGTTAATCGGGACGAATGGTCTTT<br>GTGTGAACAATGCAAAGATTTGACGGTACCGGAATAGAAAGCCACGGCTAAC<br>TACGTGCCAGCAGCCGCAGTAATACGTAGGTGGCAAGCGTTGTCCGGAATTAT<br>TGGGCGTAAAGCGCGCGCAGGCGGTTTTGCAAGTCTGTCTTAAAAGTTCGGGG<br>CTCAACCCCGTGATGGGATGGAAACTGTGGAACTAGAGTATCGGAGAGGAAA<br>GCGGAATTCCTAGTGTAGCGGTGAAATGCGTAGATATTAGGAAGAACACCAG<br>TGGCGAAGGCGGCTTTCTGGACGACAACTGACGCTGAGGCGCGAAAGCCAGG<br>GGAGCGAACGGGATTAGATACCCCGGTAGTCCTGGCCGTAAACGATGGGTAC<br>TAGGTGTAGGAGGTATCGACCCCCTTTGTGCCGGAGTTGACGCAATAAGTACC<br>CCGCCTGGGGAGTACGGTCGCACGGGTGAAACTCTAAGGAATTGACGGGGGC<br>CCGCACACGCGGTGGAGTACGTGGTTTAATTTGACGCAACGCGAAGAGCCCTT<br>CCAGGTCTCGACACTGATGGACGAAACAAGAGATTGTTTTTCTCCCTCGGGAG<br>CCAGAAAACAGGTGGTGCACGGCTGTCGTCAGCTCGTGTCGTGAGATGTTGGG<br>TTAAGTCCCGCAACGAGCGCAACCCCTATCTTATGTTGCCAGCACTTCGGGTG<br>GGAACTCATGAGAGACTGCCGCAGACAATGCGGAGGAAGGCGGGGATGACG<br>TCAAGTCATCATGCCCCTTATGACCTGGGCTACACACGTACTACAATAGGCTT<br>TAATAGAGGGACGCGAAGCCGCGAGGTGGAGCAAACCCCAGAAACAAGCTCT<br>CAGTTCGGATCGTAGGCTGCAACTCGCCTACGTGAAGTCGGAATCGCTAGTAA<br>TCGCAGGTCAGCATACTGCGGTGAATACGTTCCCGGGCCTTGCACACACCGCC<br>CGTCA |

The major OTUs were arranged in order of abundance at each dietary animal protein medium.

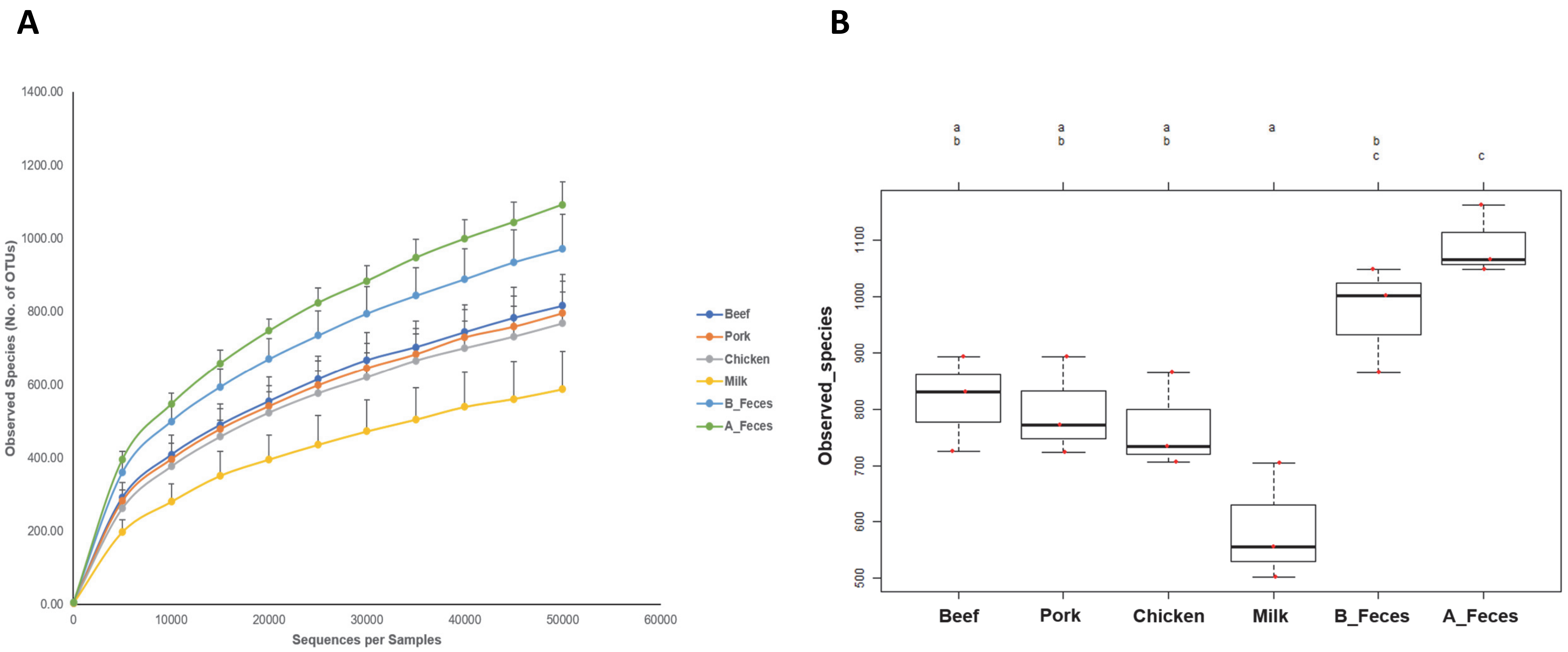


**Figure S1**. Rarefaction analysis observed species (No. of OTUs) from the different animal protein groups. (A) The lines indicate the mean and the error bars represent standard deviations. Colors mean: *blue* Beef, *orange* Pork, *gray* Chicken, *yellow* Milk, *sky blue* B_Feces, and *green* A_Feces. (B) This graph shows randomly selected OTUs in 50000 reads. Red dots mean each sample.  a, b , c represents that difference is significant at 0.05 level.

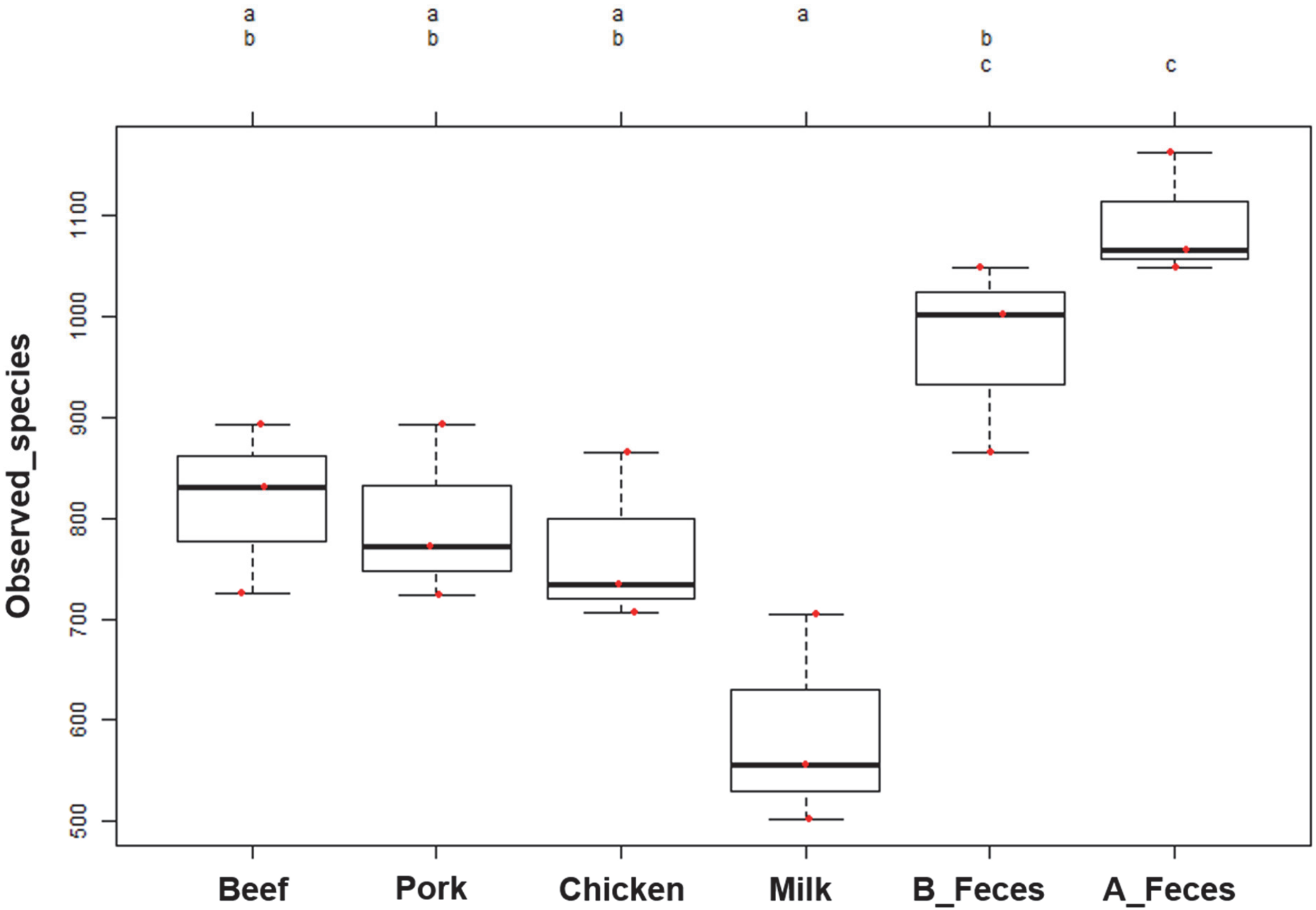


**Figure S2**. Rarefaction analysis observed species (No. of OTUs) from the different animal protein groups. This graph shows randomly selected OTUs in 50000 reads. Red dots mean each sample. a, b , c represents that difference is significant at 0.05 level.
